# Supplementary figures and images for: Relationships within Bolbitis sinensis Species Complex Using RAD Sequencing
Source: Plants (Basel). 2024 Jul 20;13(14):1987. doi: 10.3390/plants13141987 (PMC11280518; doi:10.3390/plants13141987)

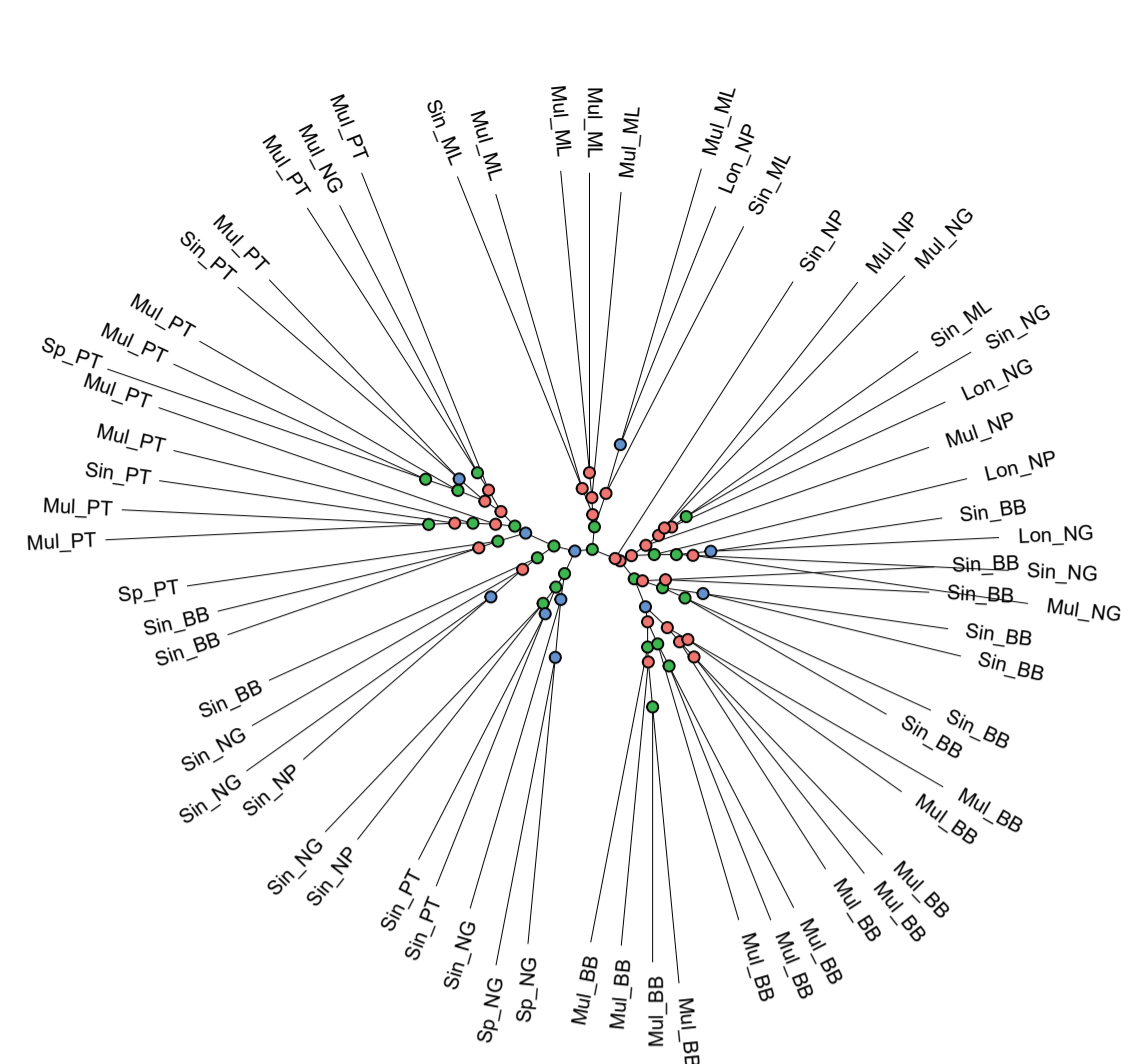

p1-r0.30 26,955

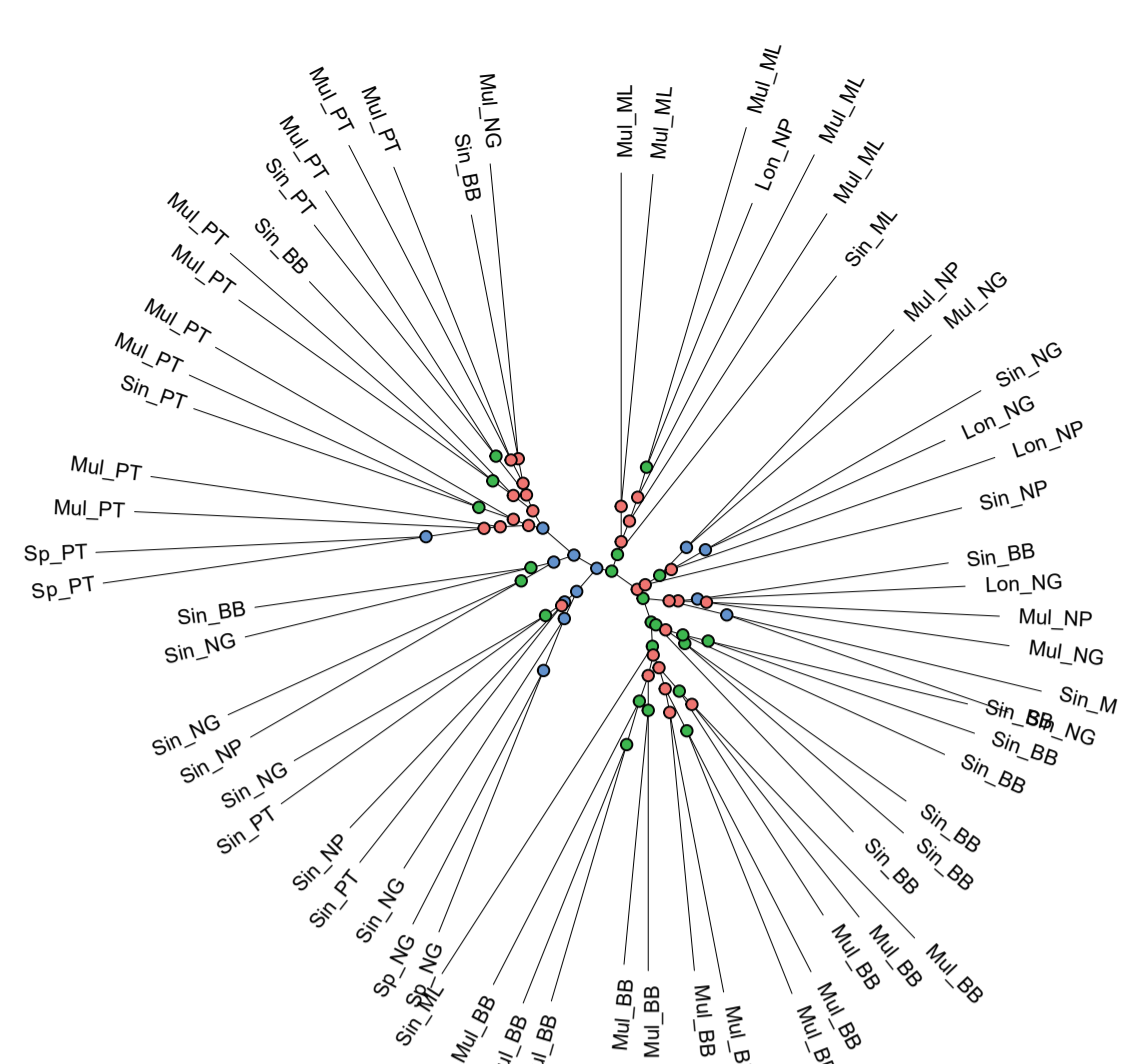

p1-r0.40 17,475

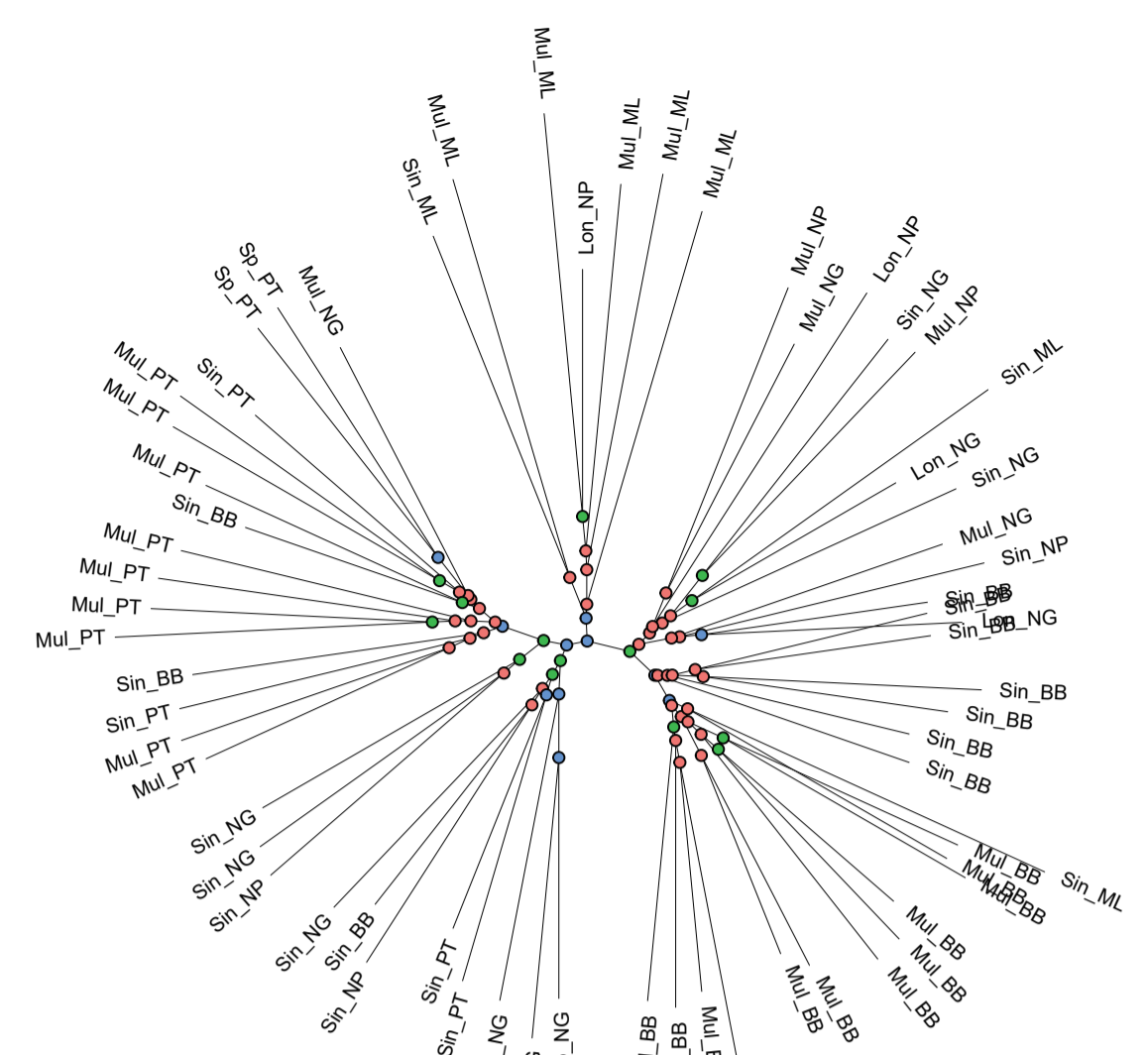

p1-r0.50 12,774

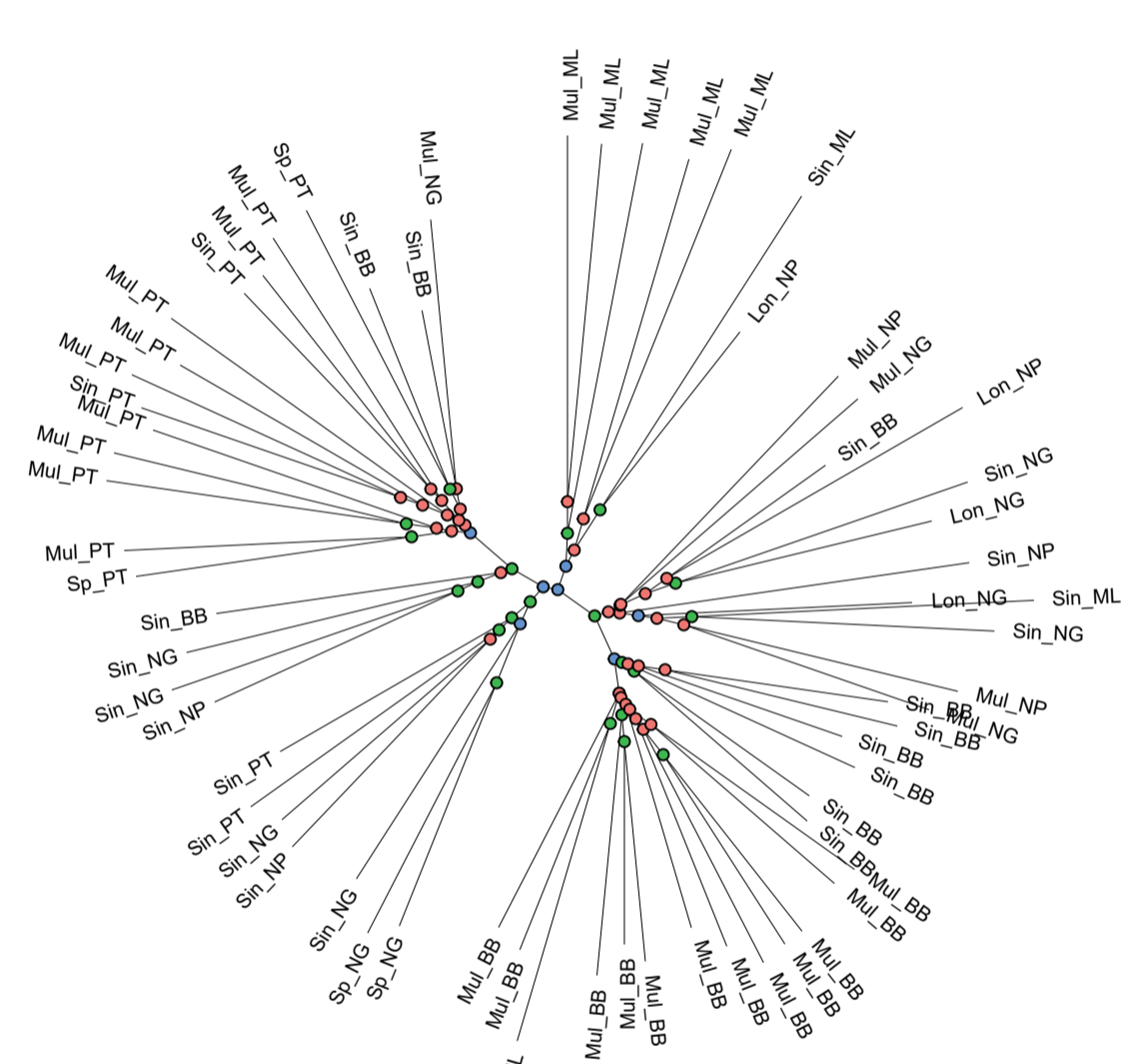

p1-r0.60 7,771

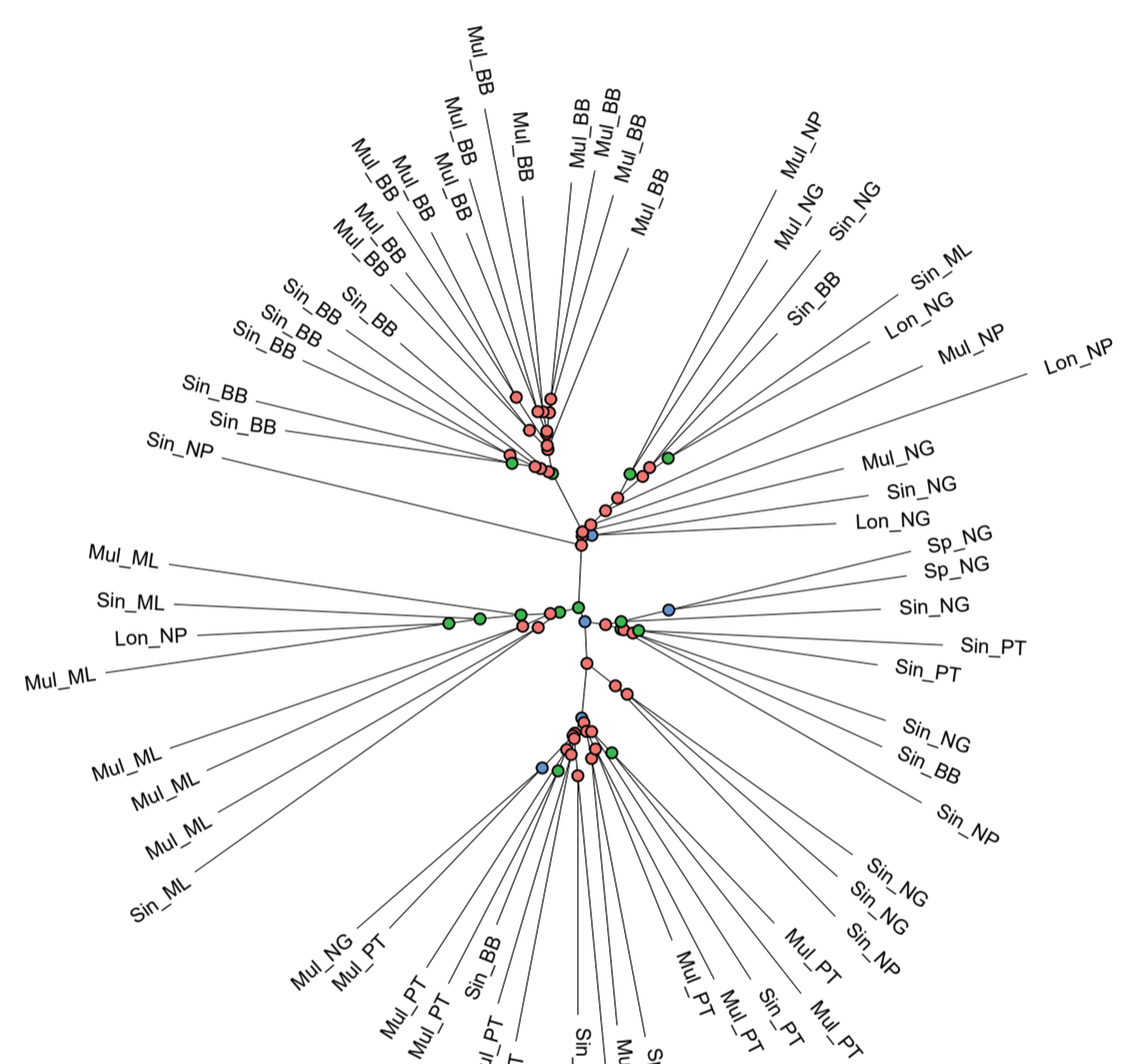

p1-r0.70 4,671

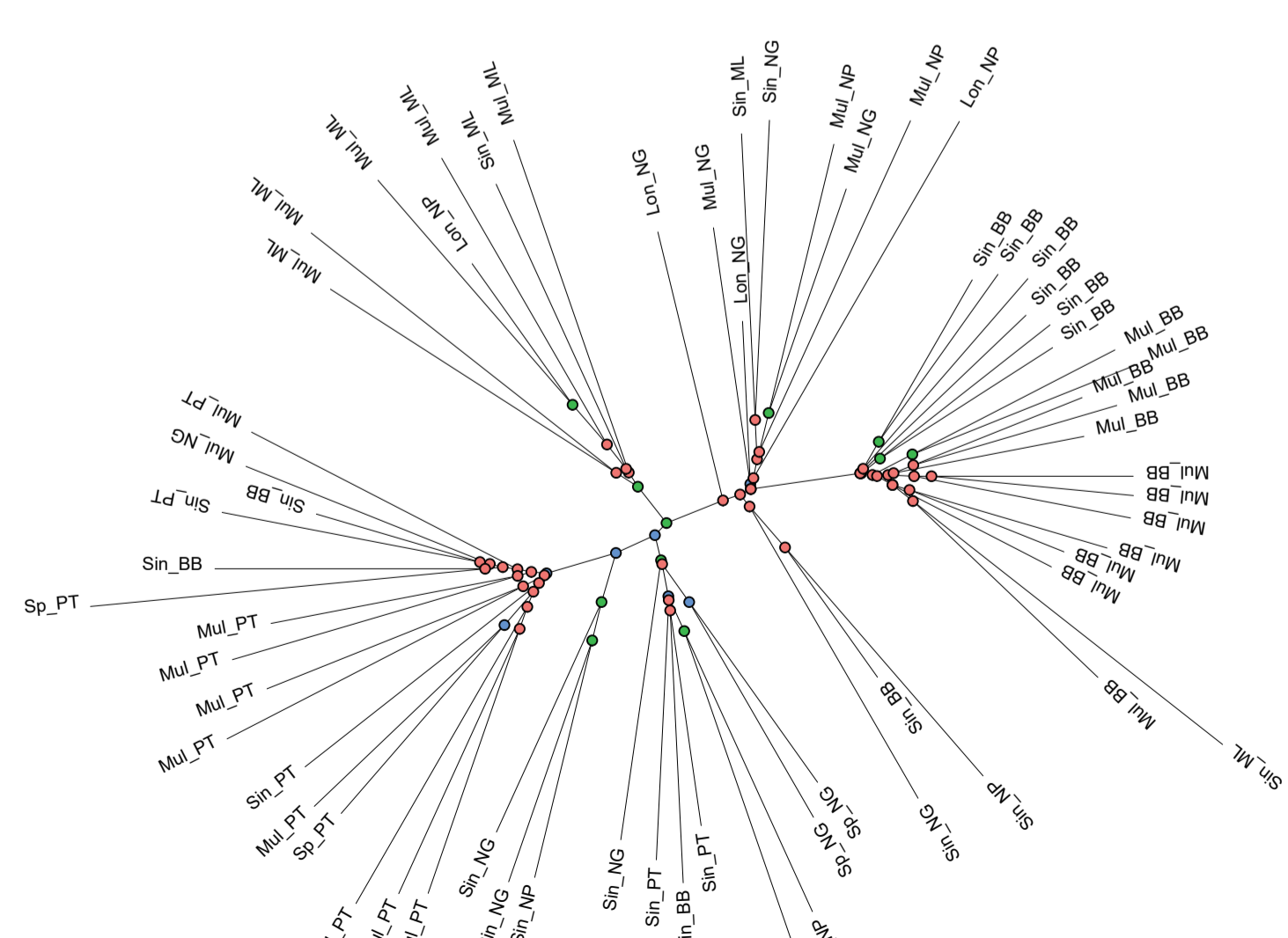

p1-r0.80 2,688

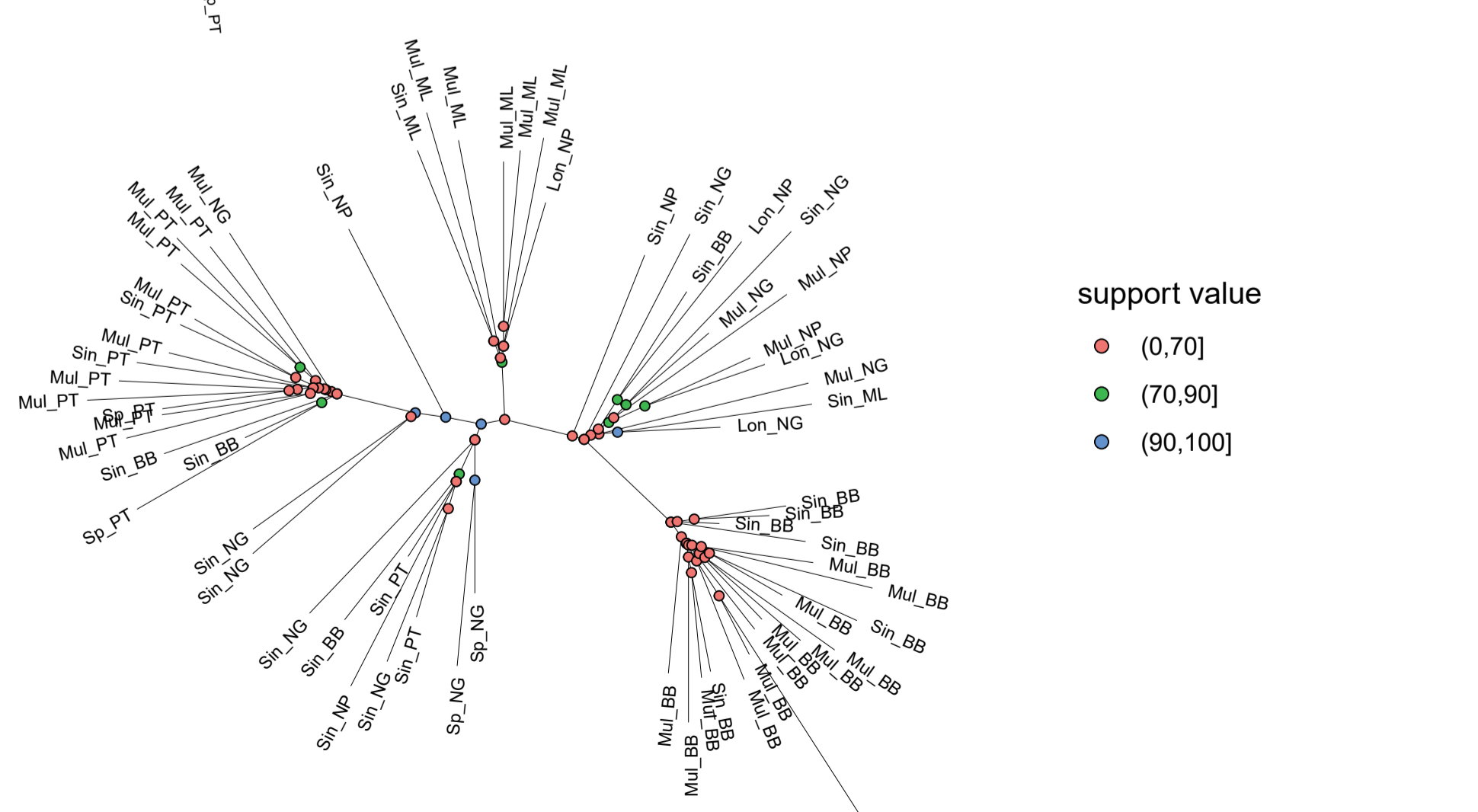

p1-r0.90 1,143

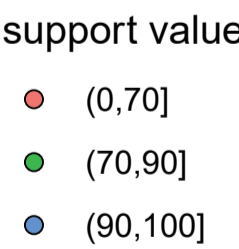

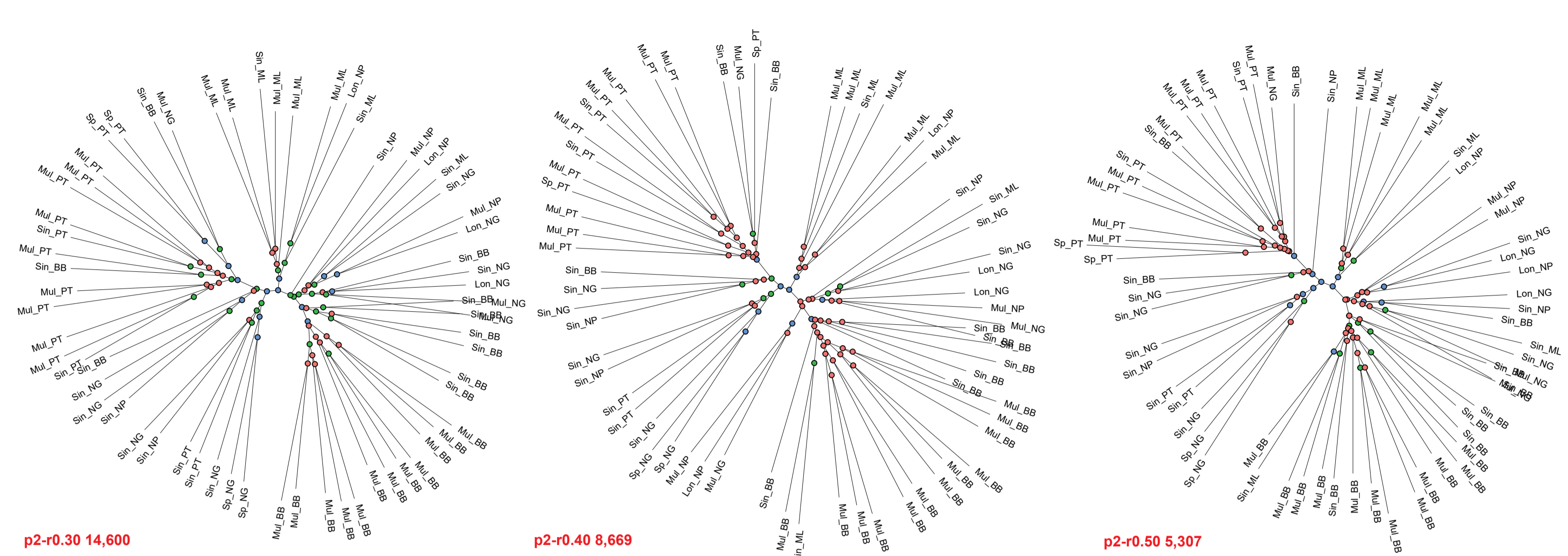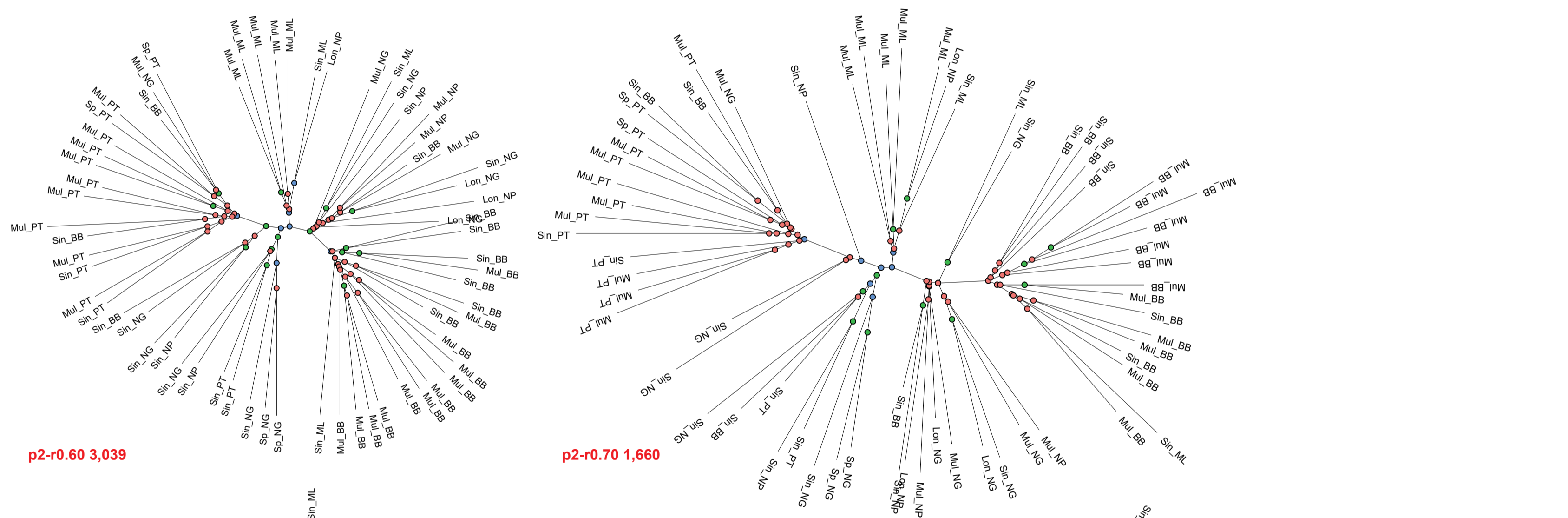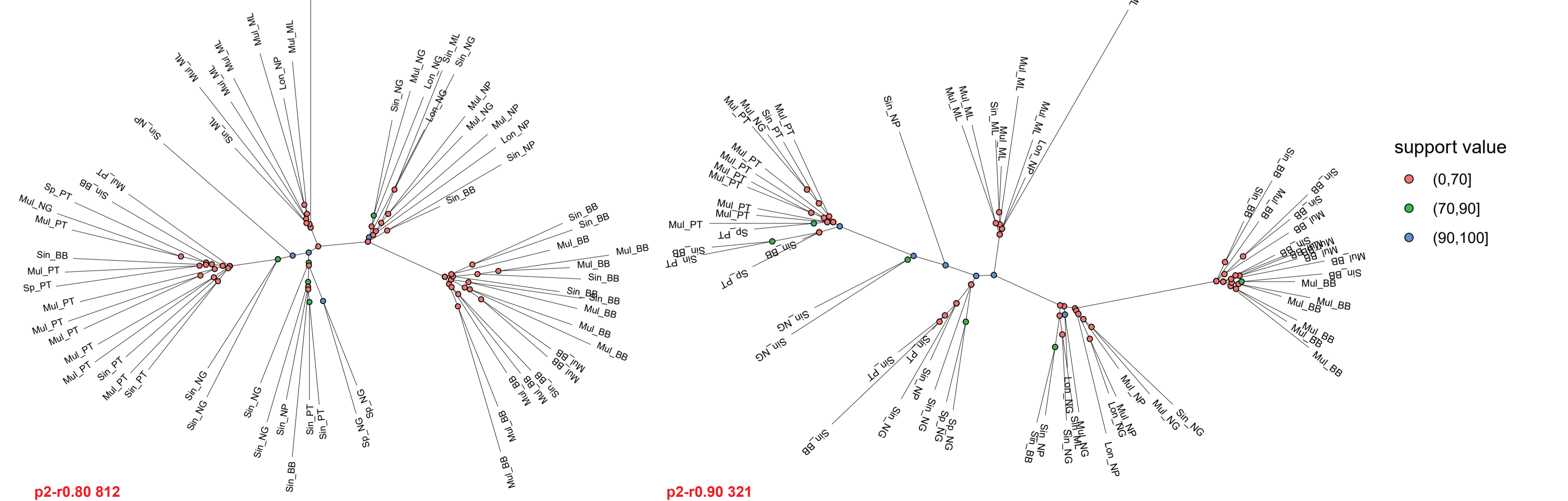

support value

- (0,70]
- (70,90]
- (90,100]

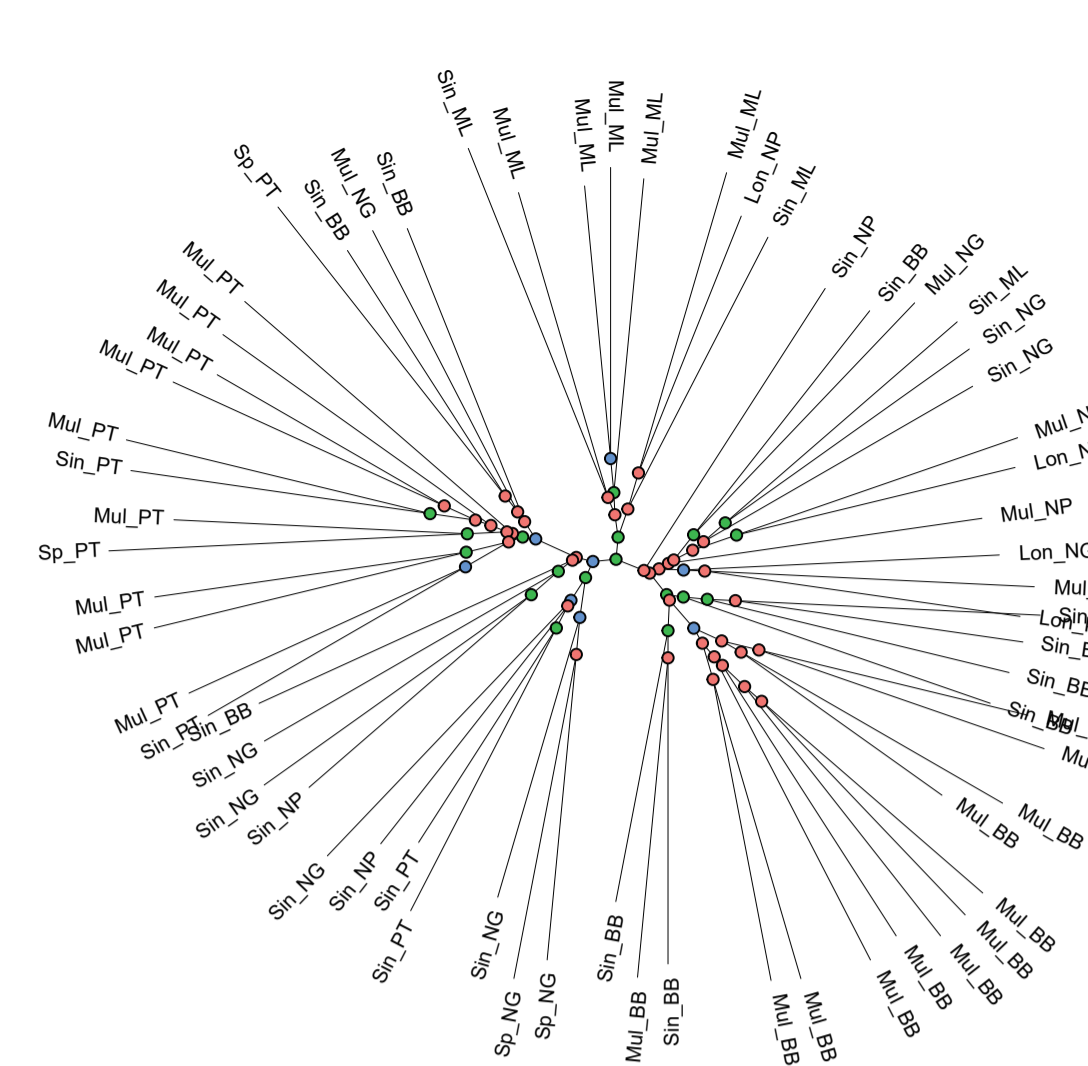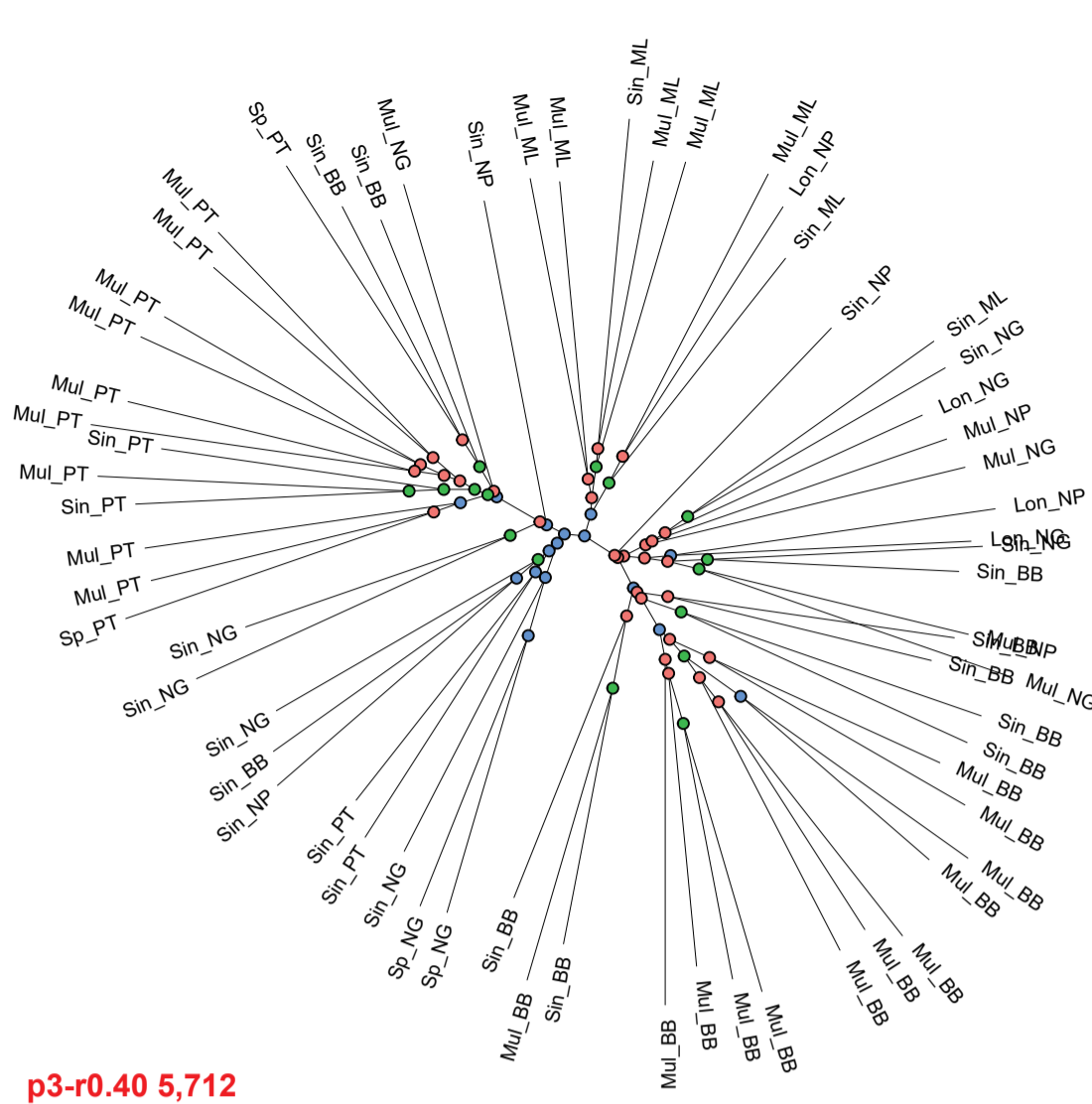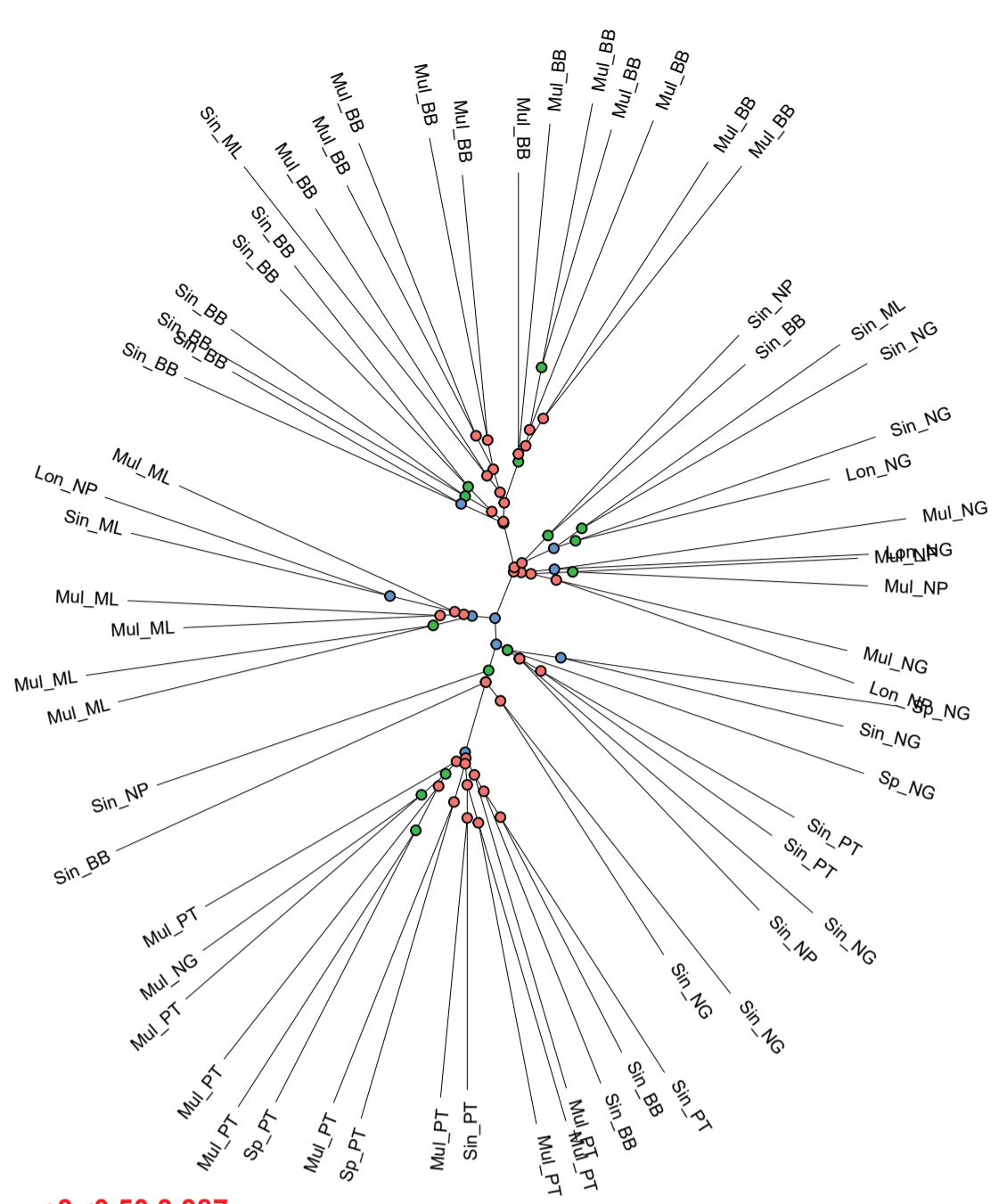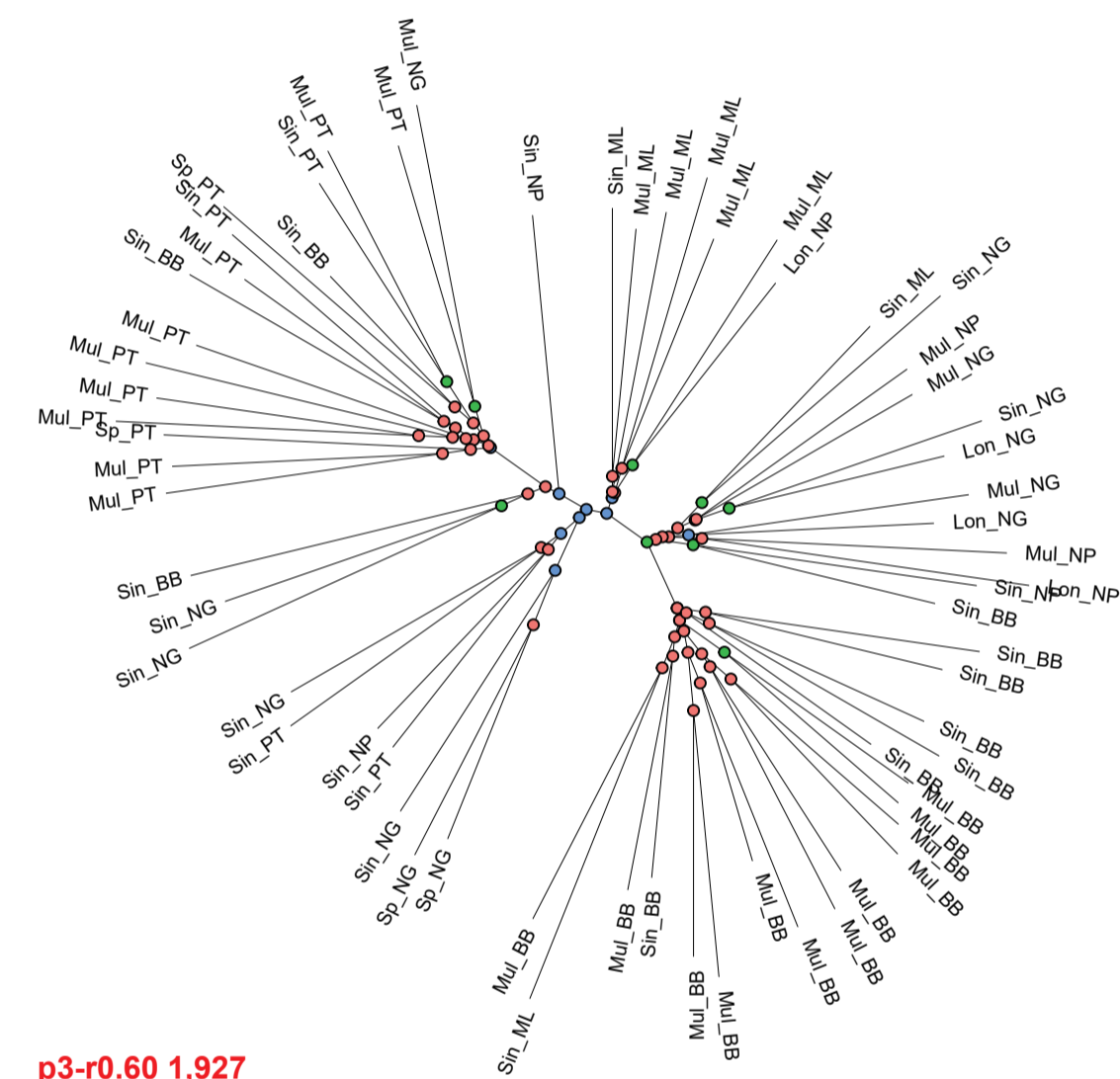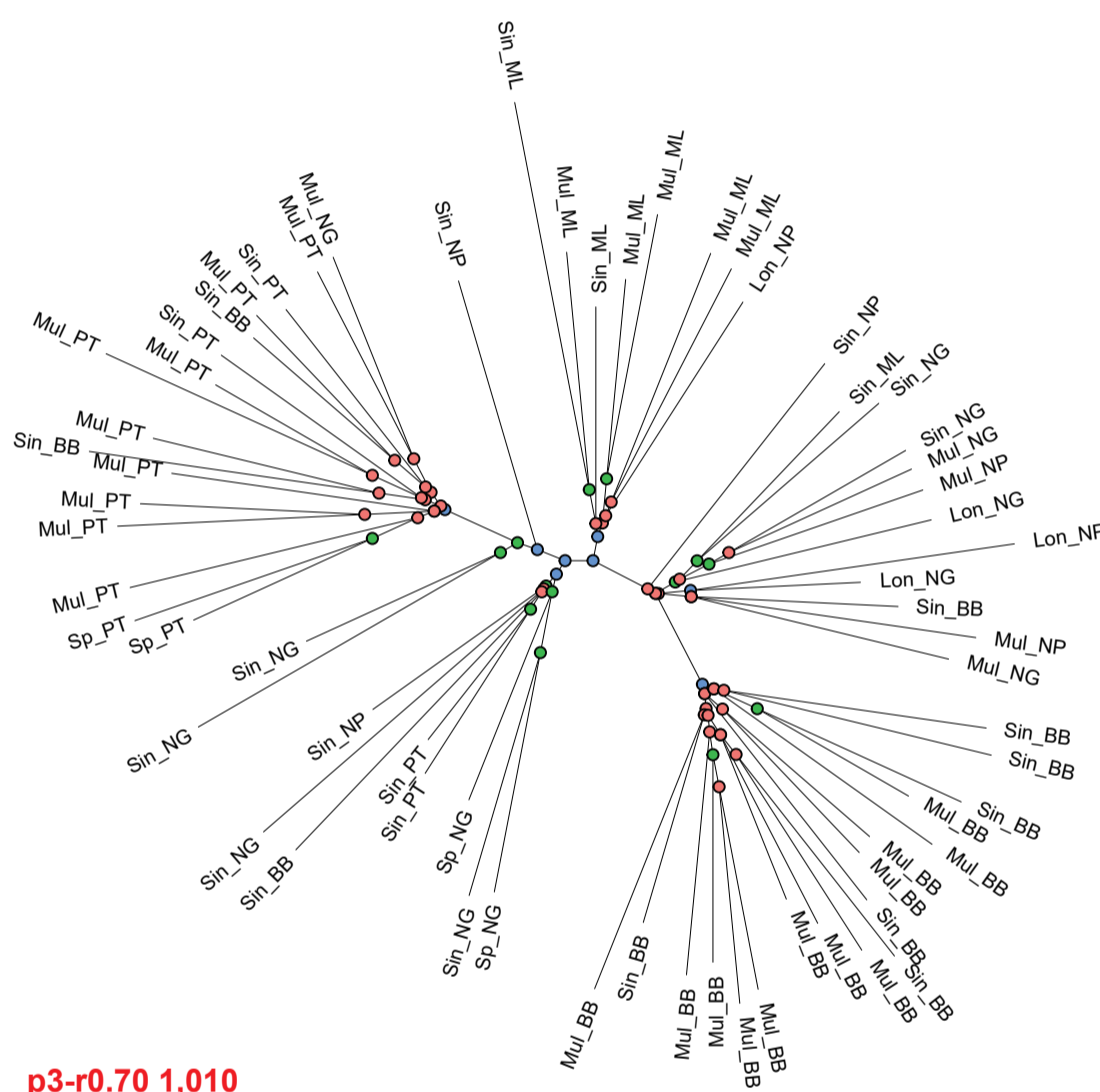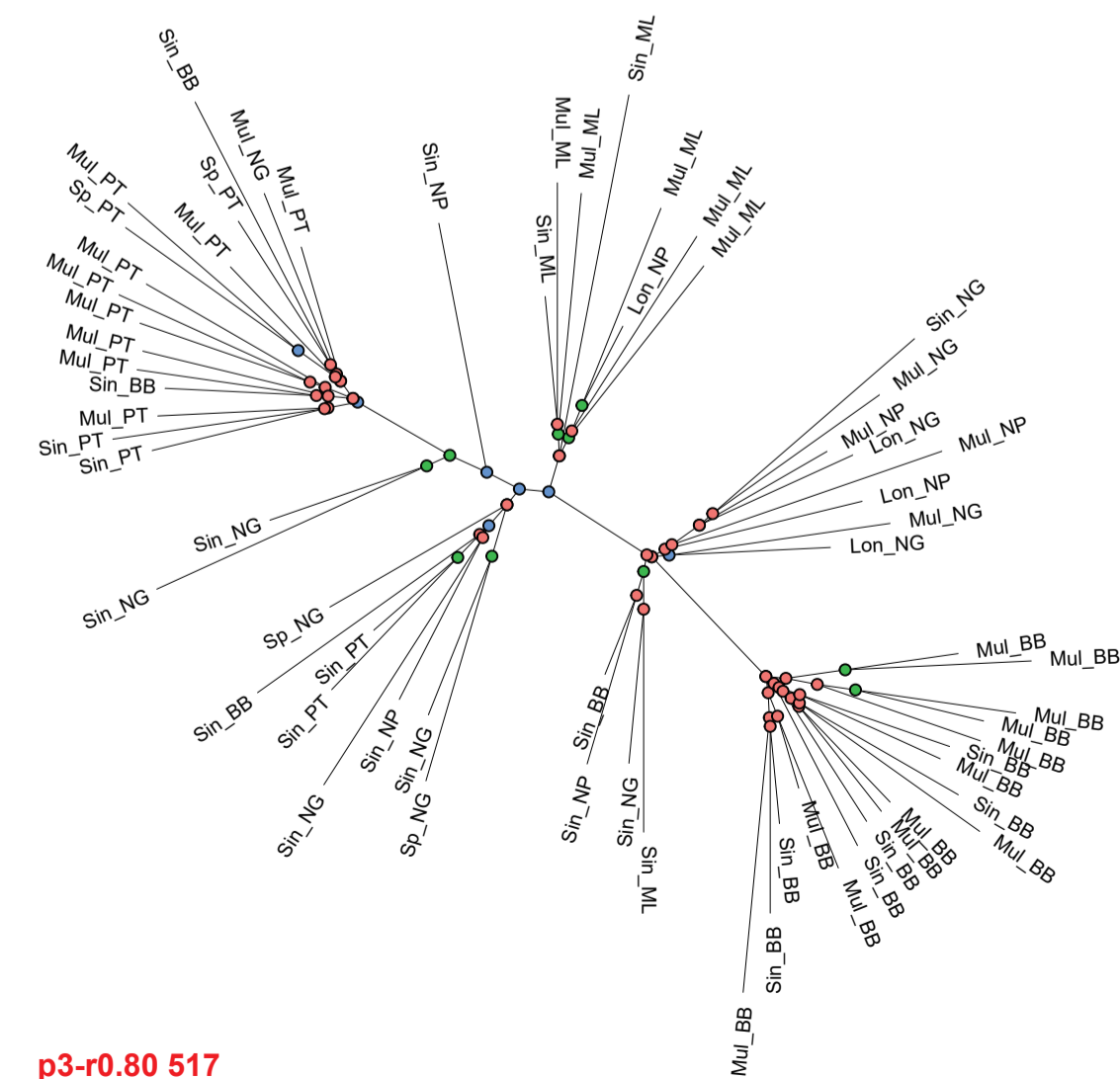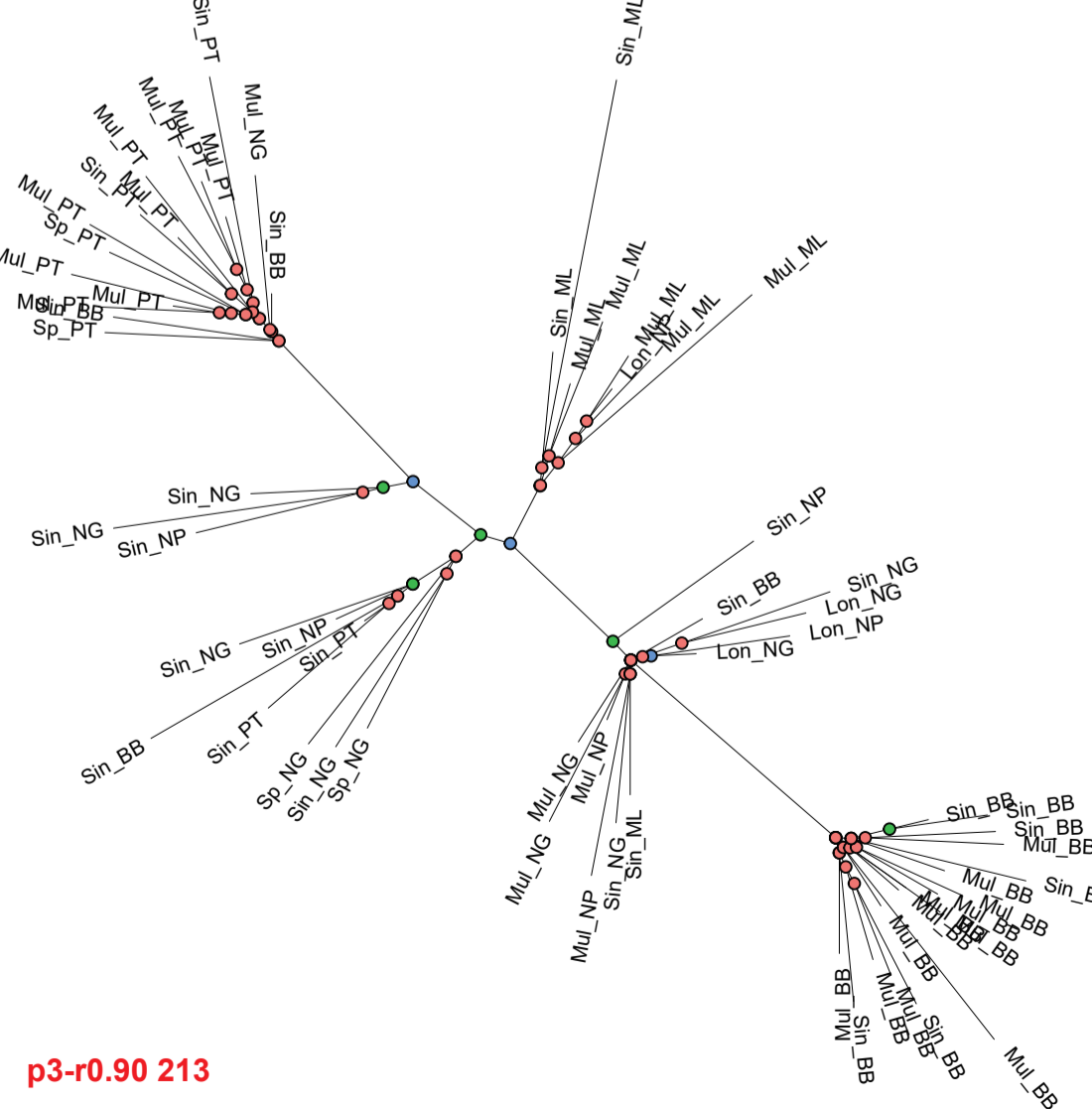

support value

- $(0,70]$

- $(70,90]$

- $(90, 100]$

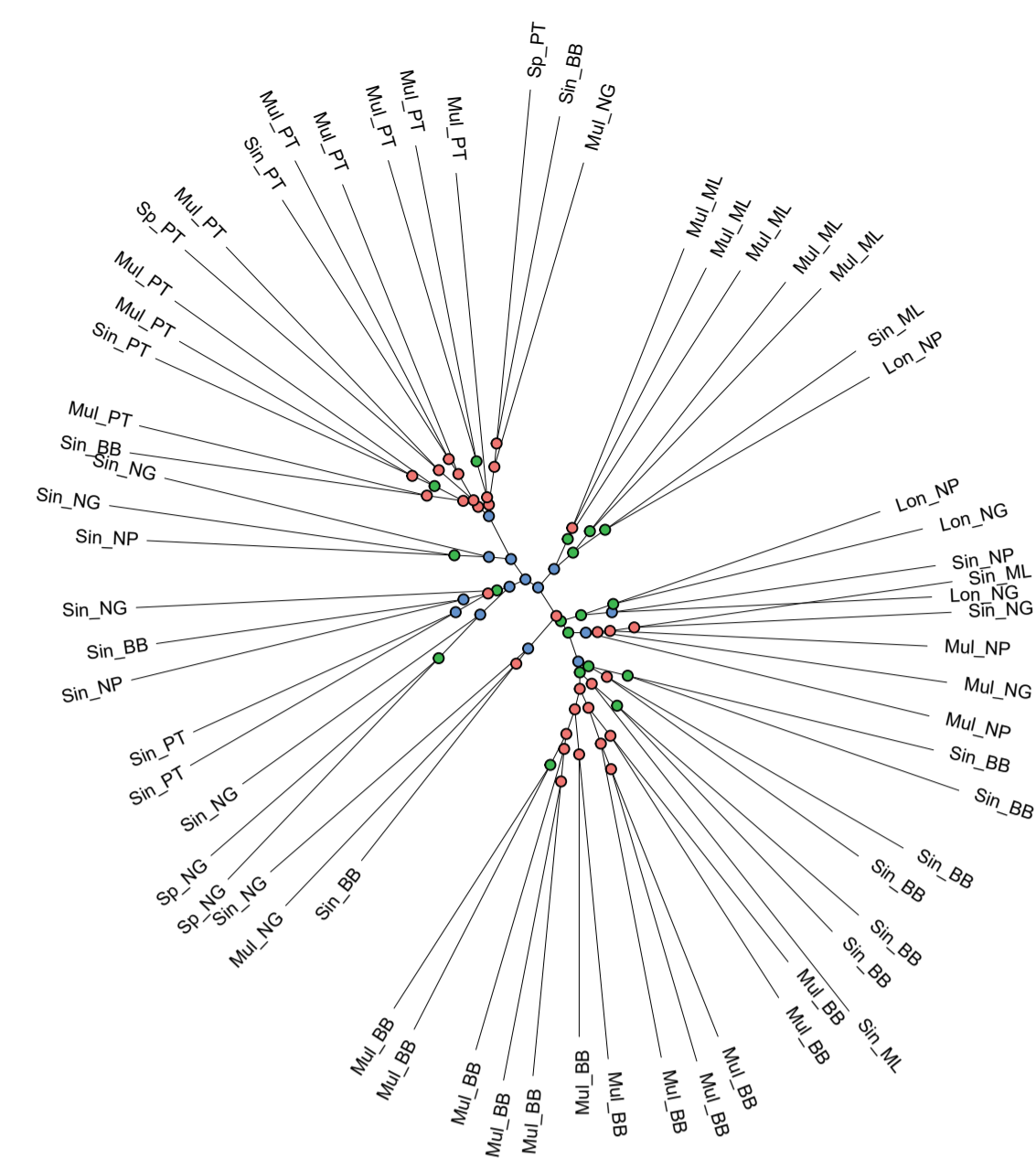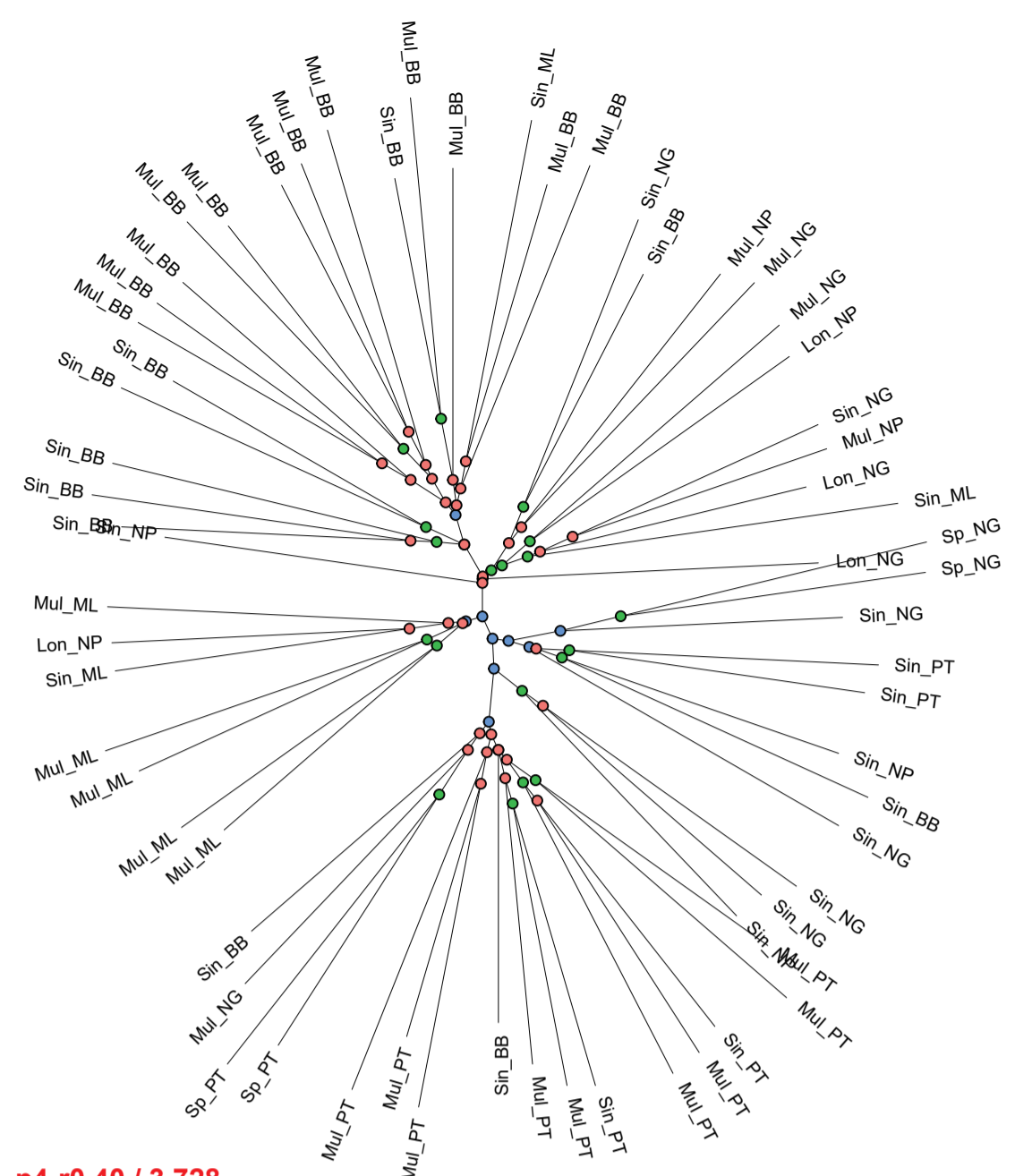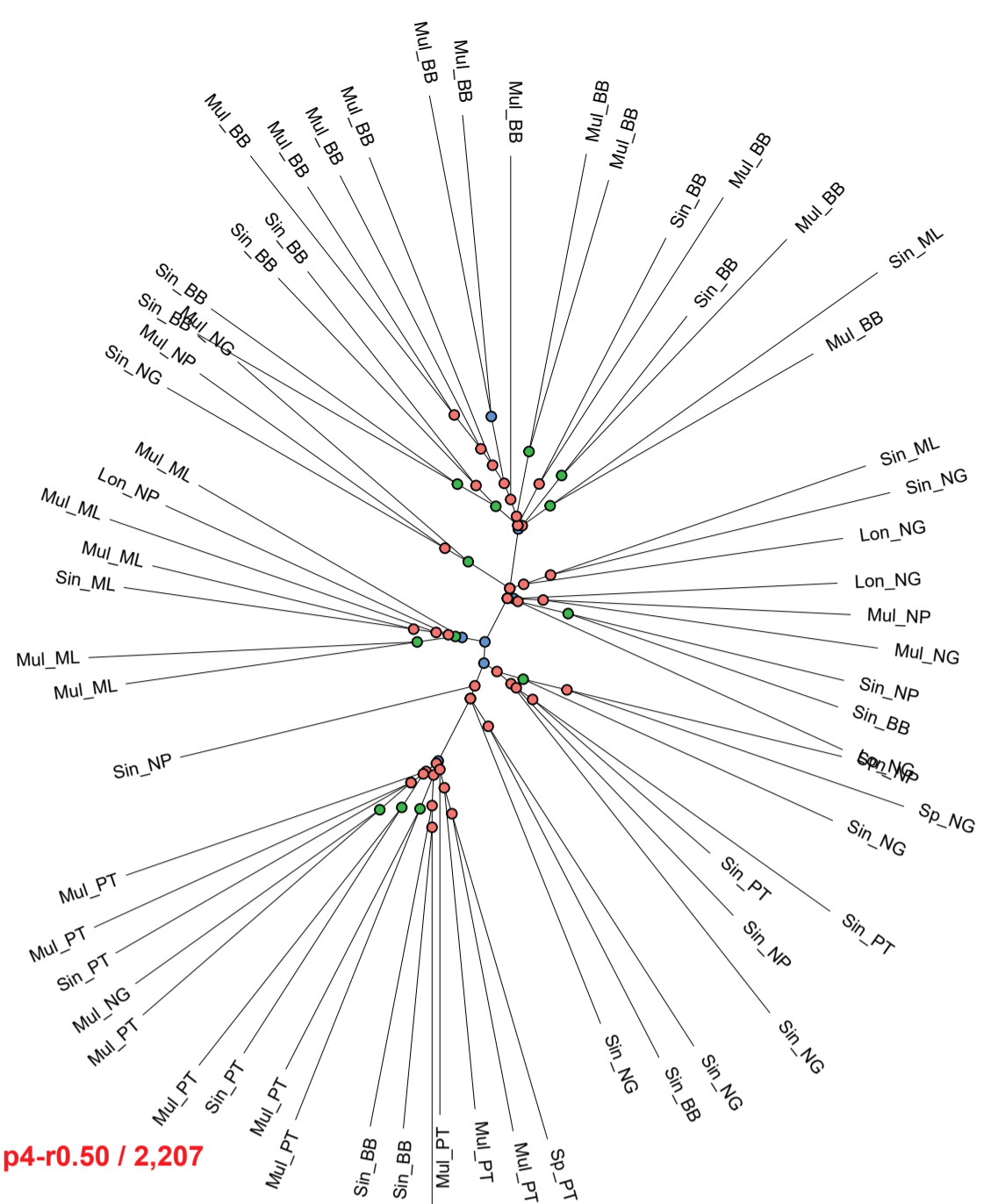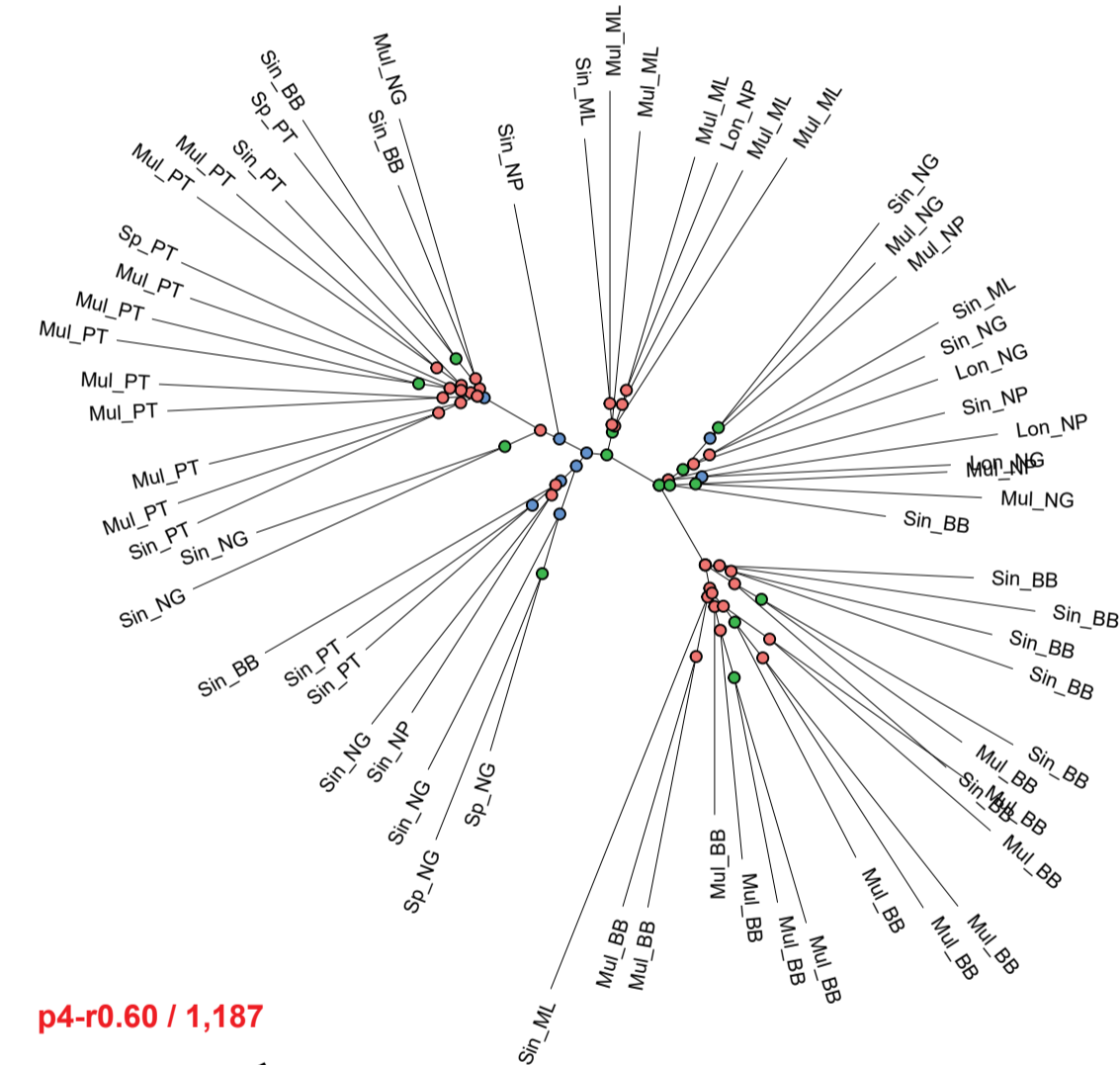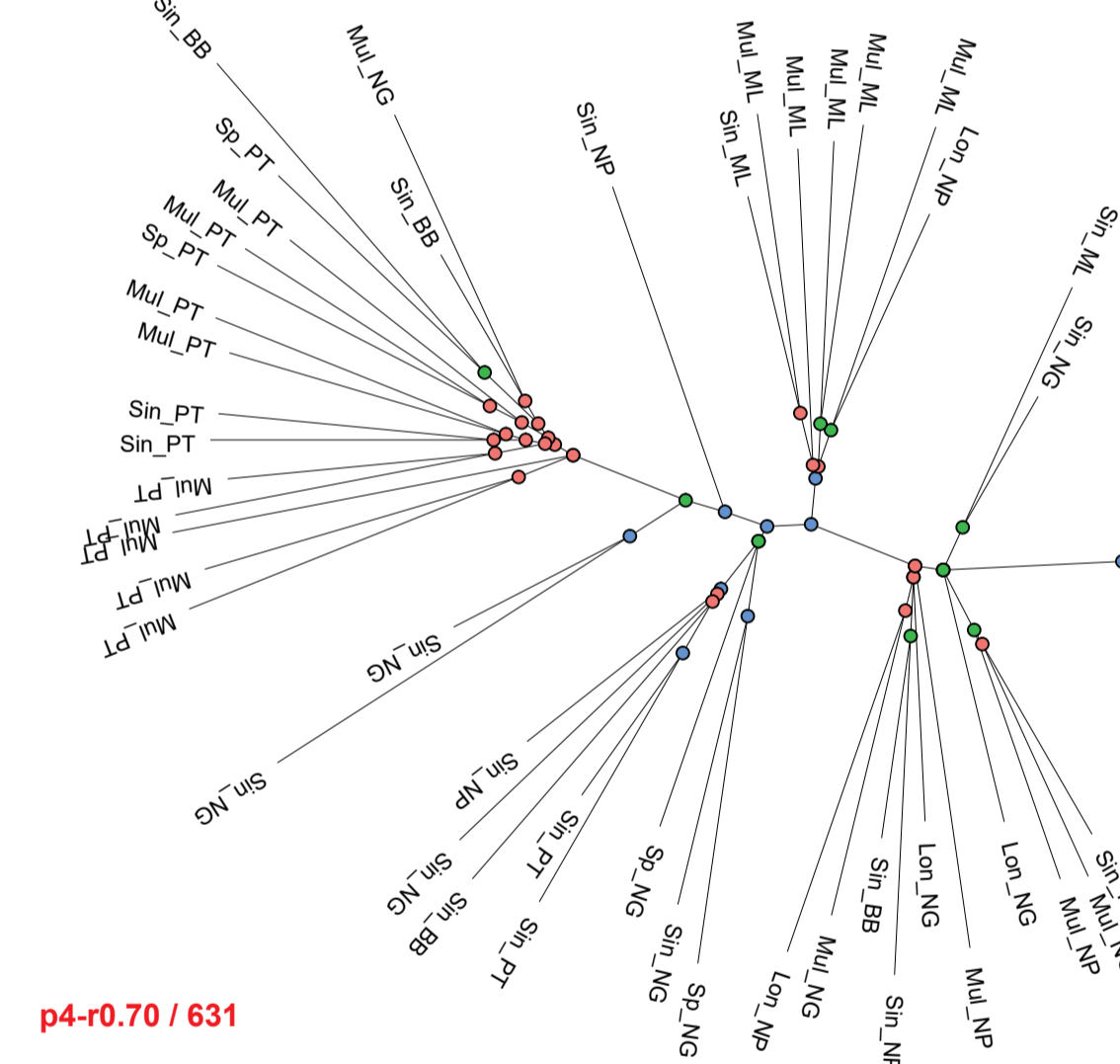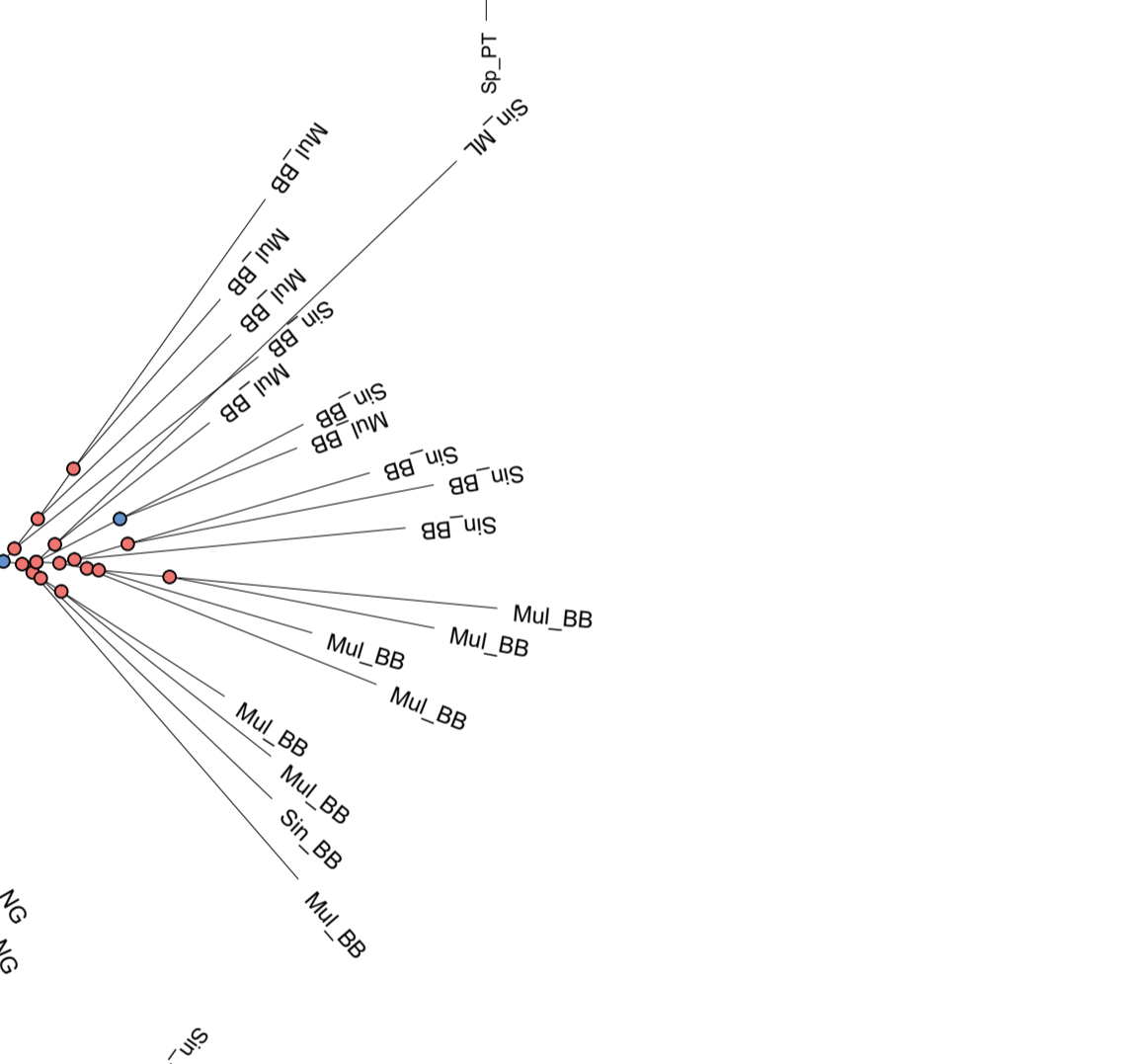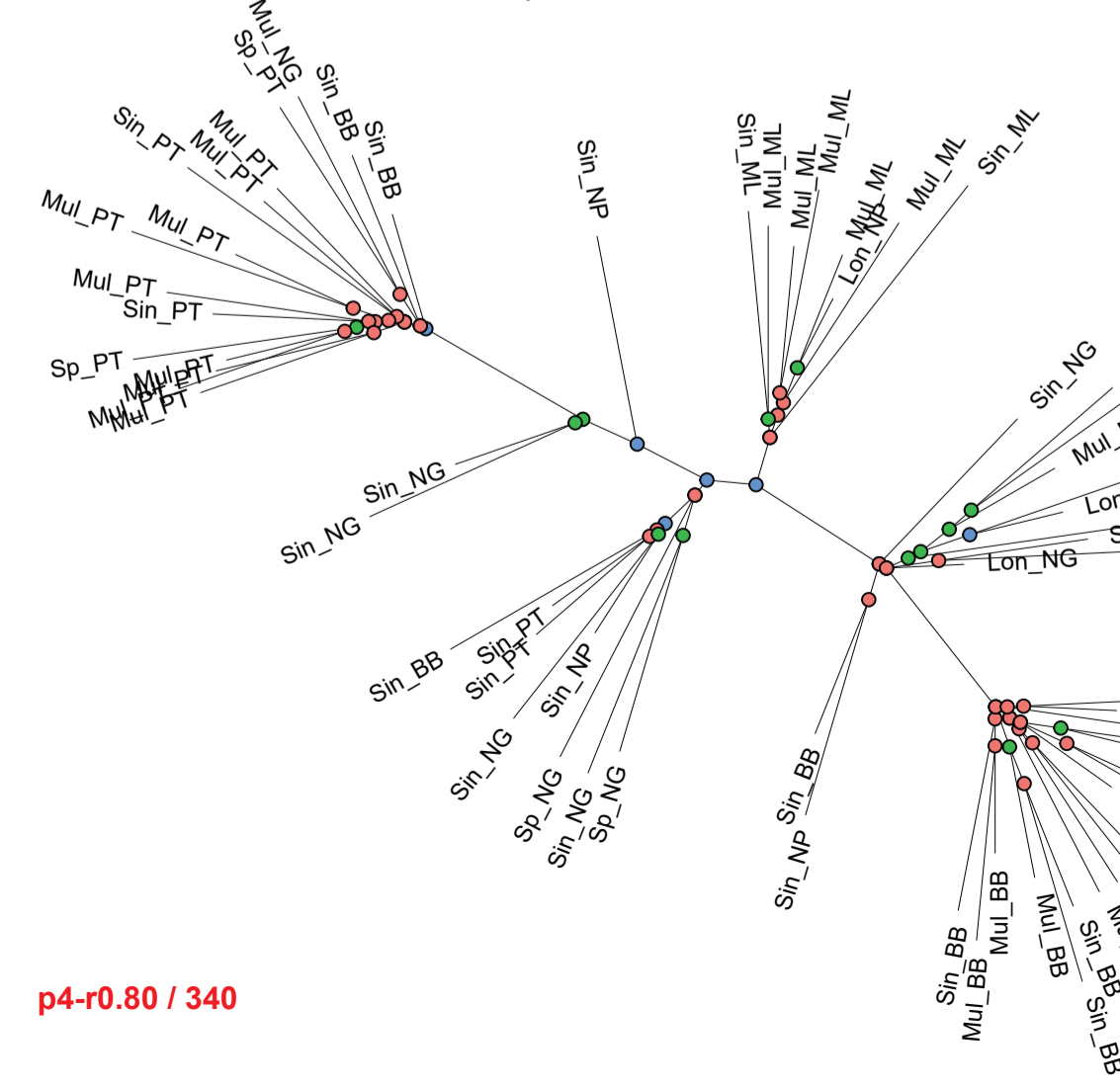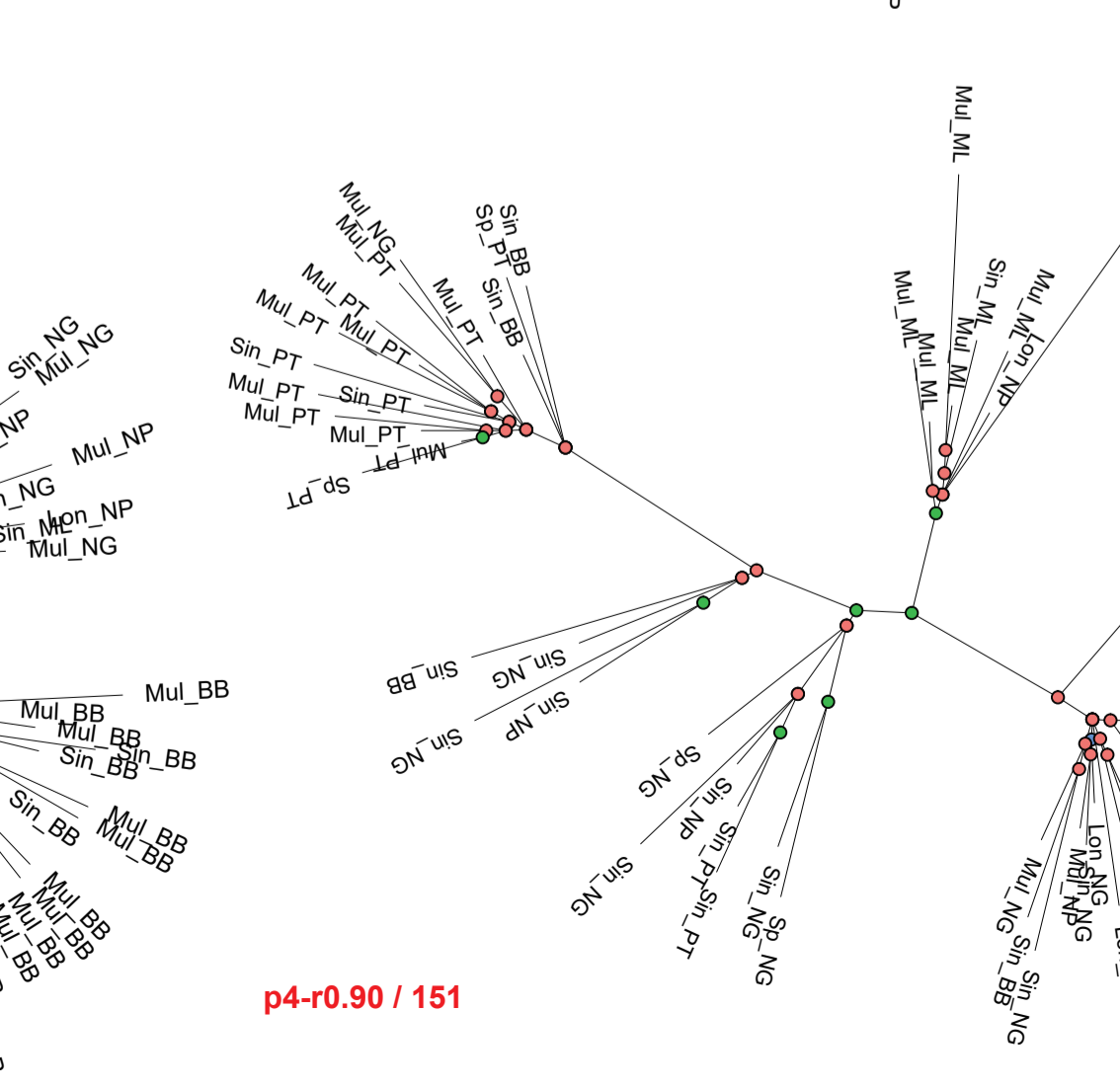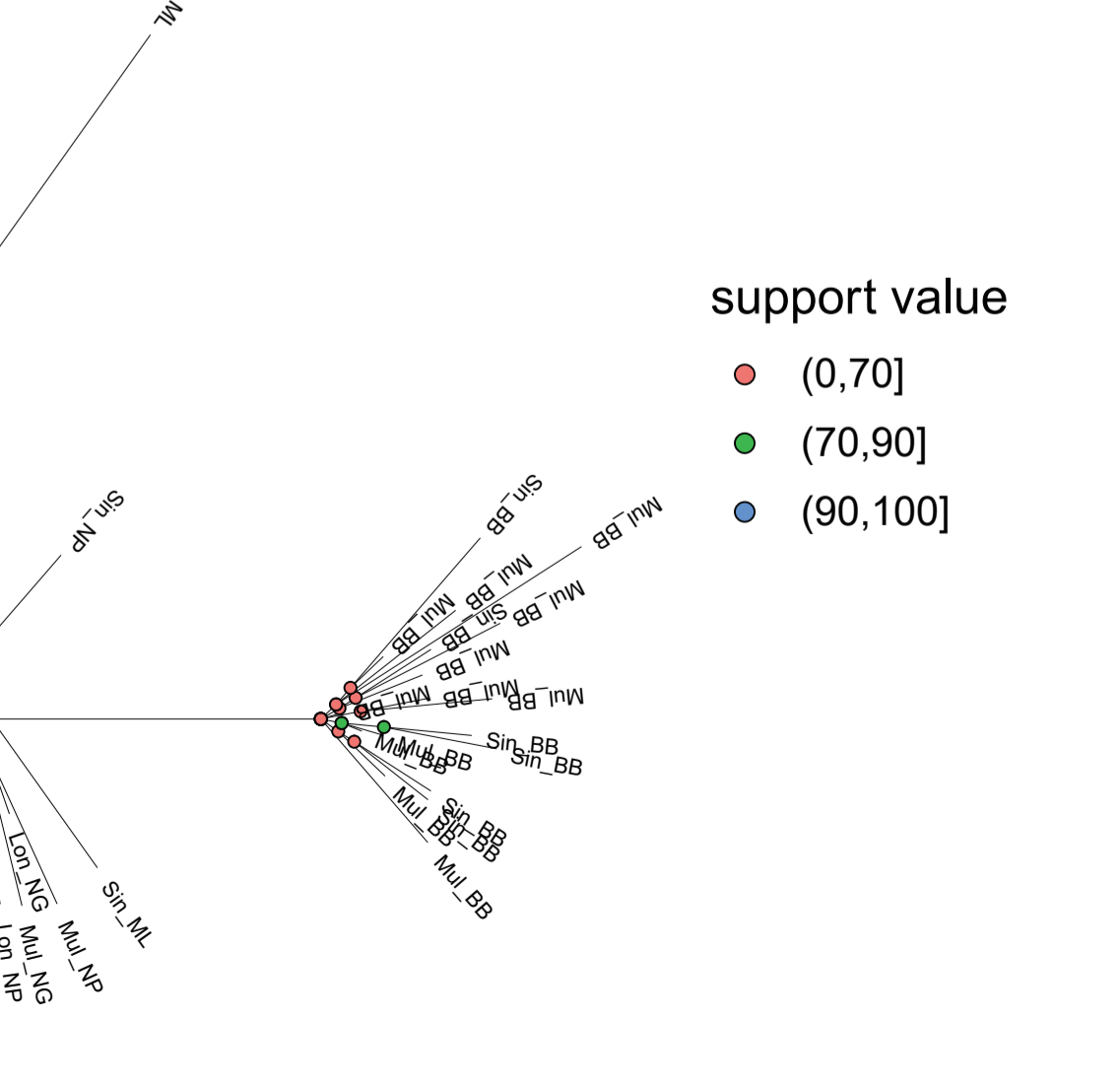

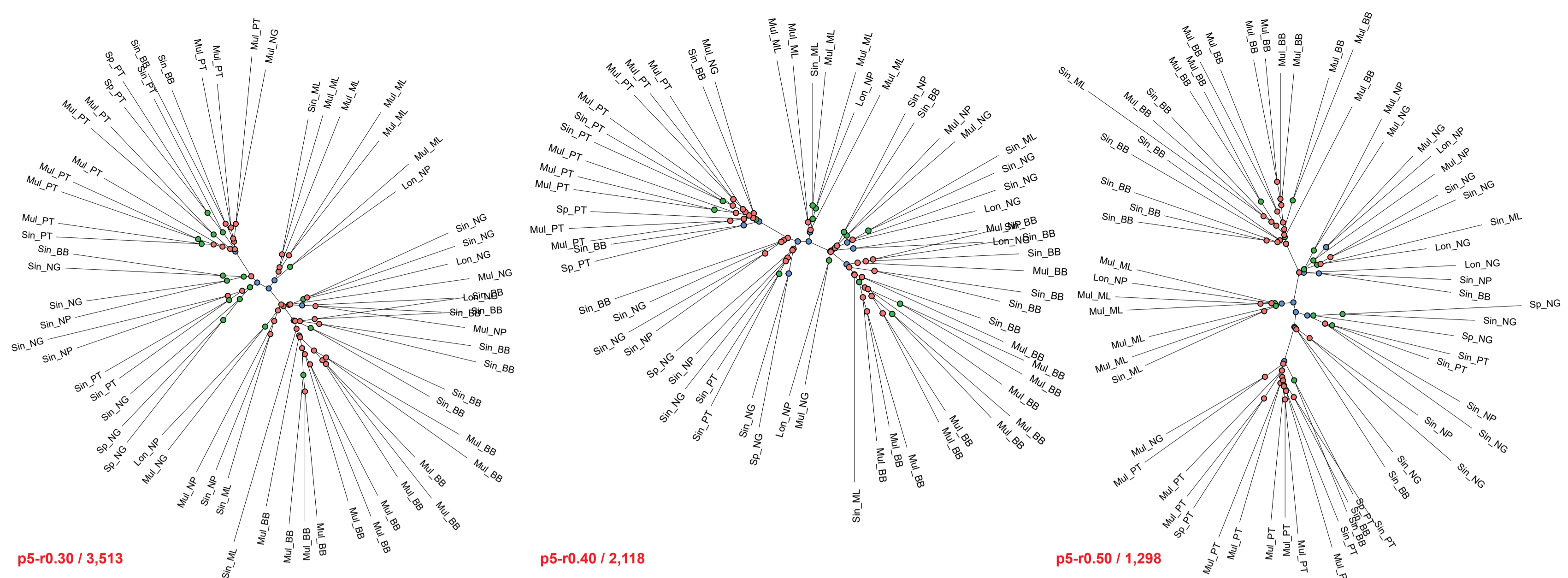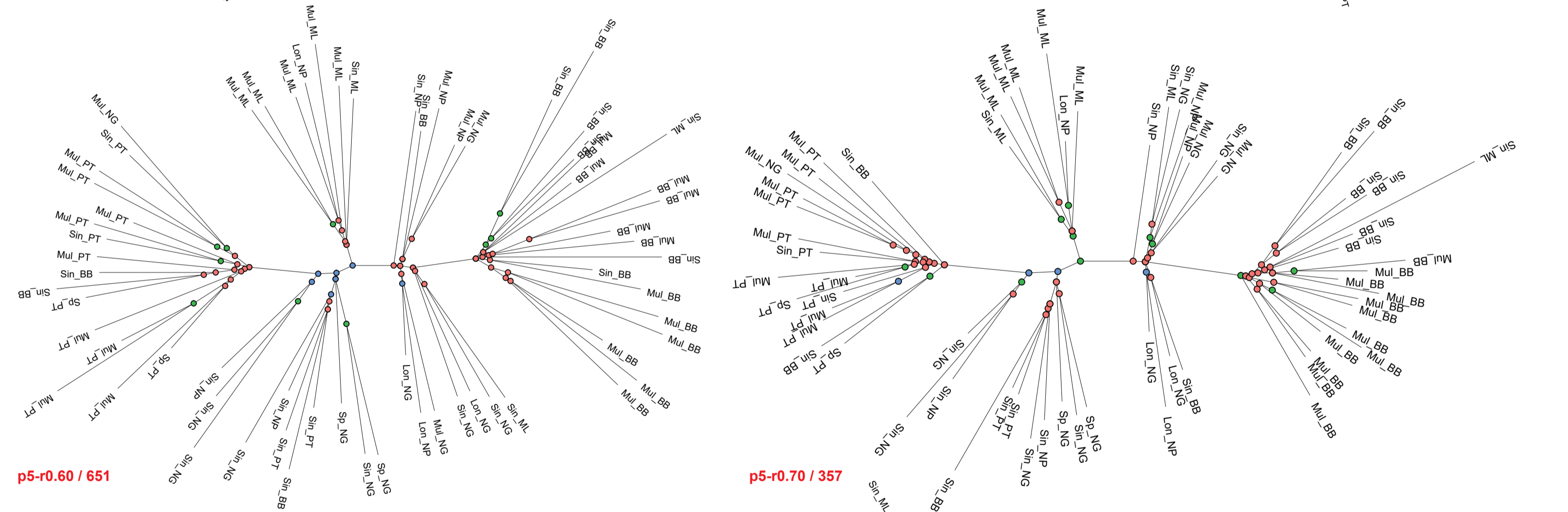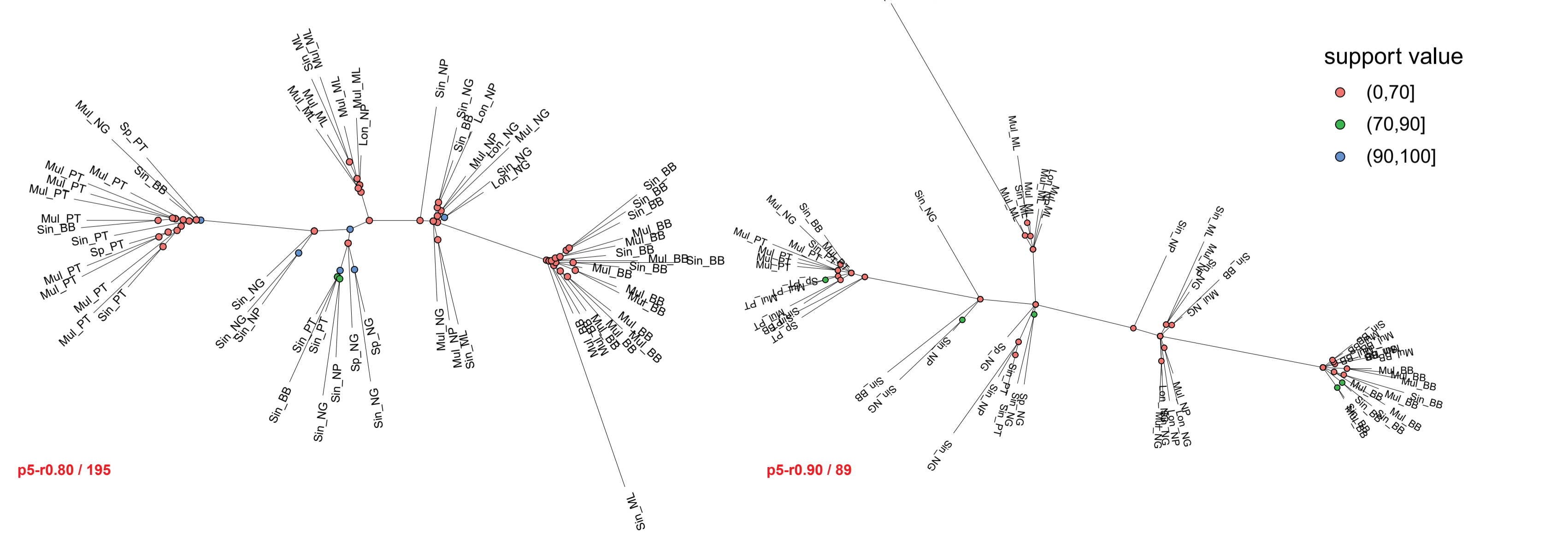

support value

(0,70]

(70,90]

(90,100]

Supplement: Supplementary file 1 [file plants-13-01987-s001.zip › Figure S1_MLtree-merged.pdf]

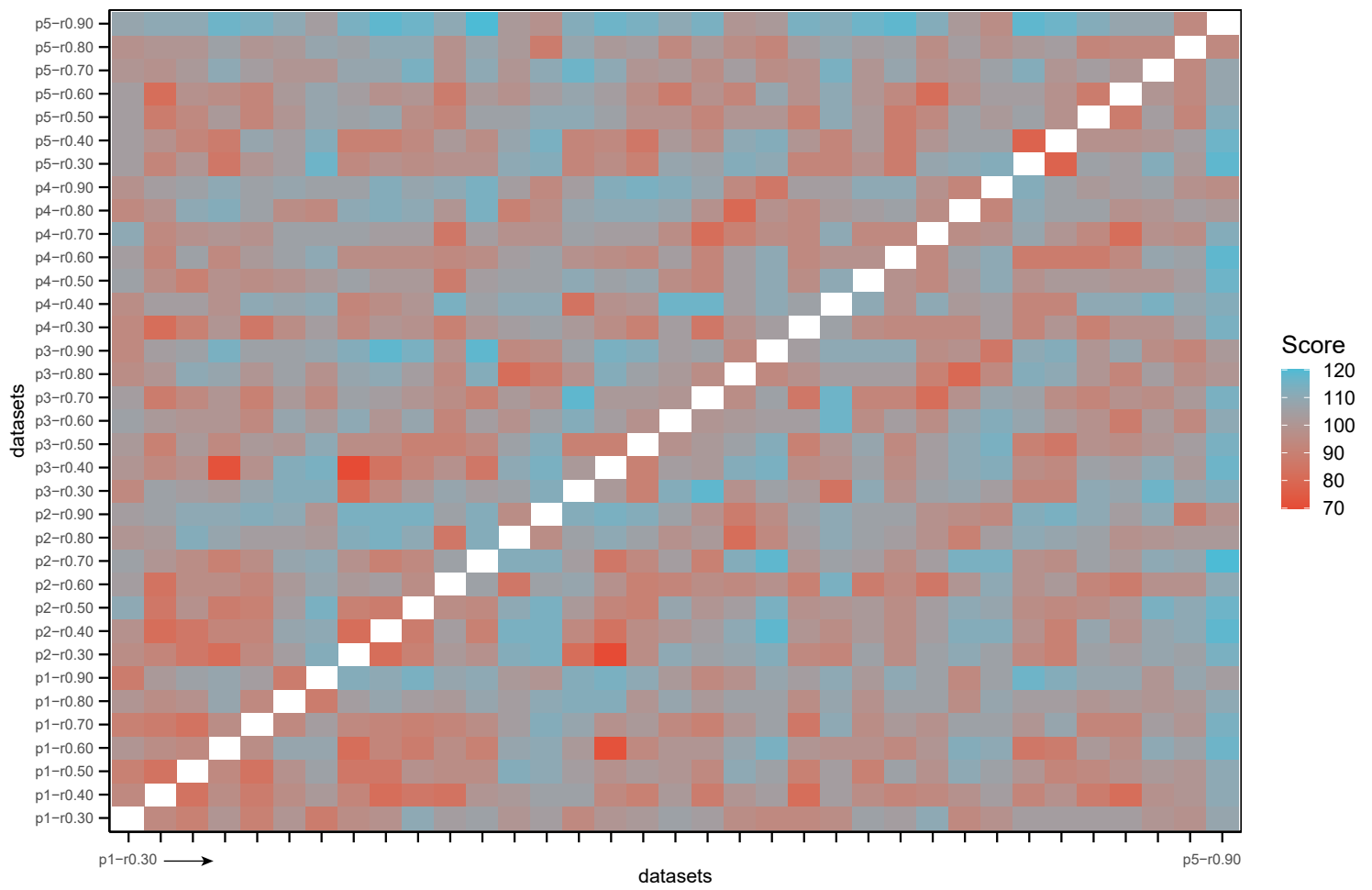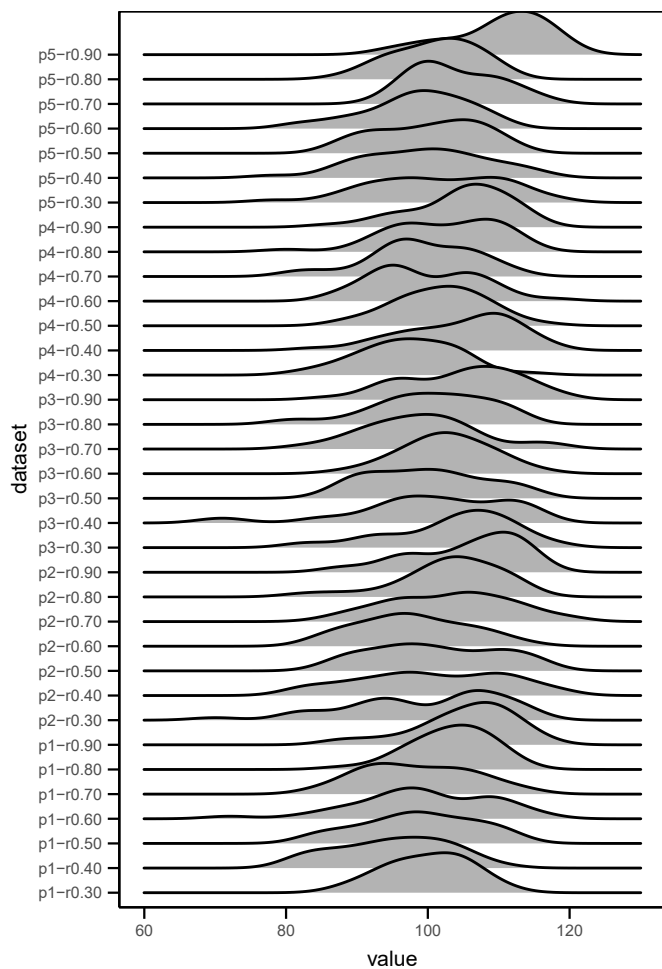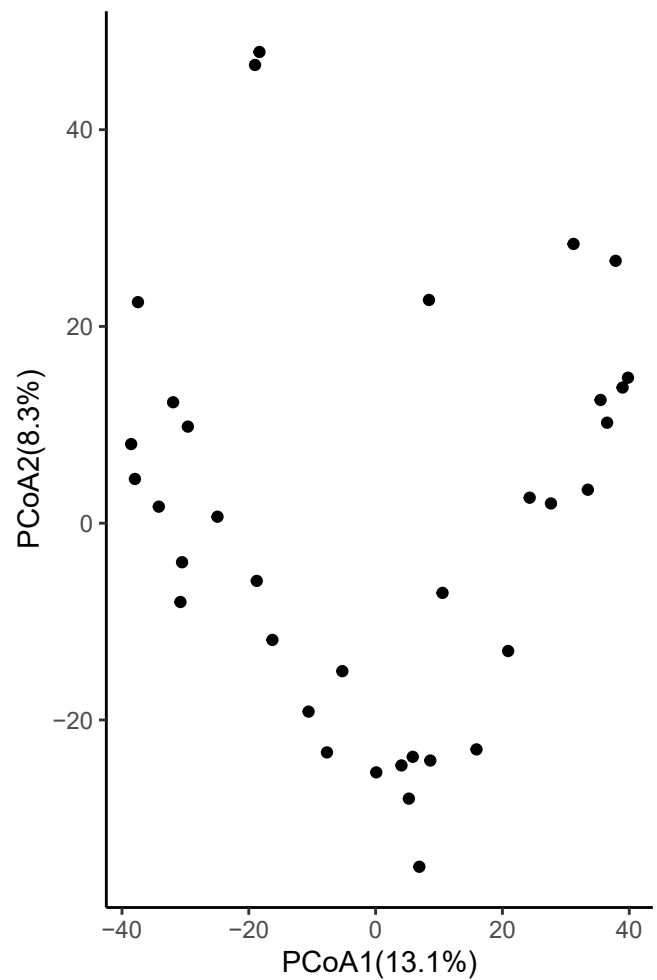

Supplement: Supplementary file 1 [file plants-13-01987-s001.zip › Figure S2_distribution, heatmap, and PCoA plot based on RF distance between trees.pdf]

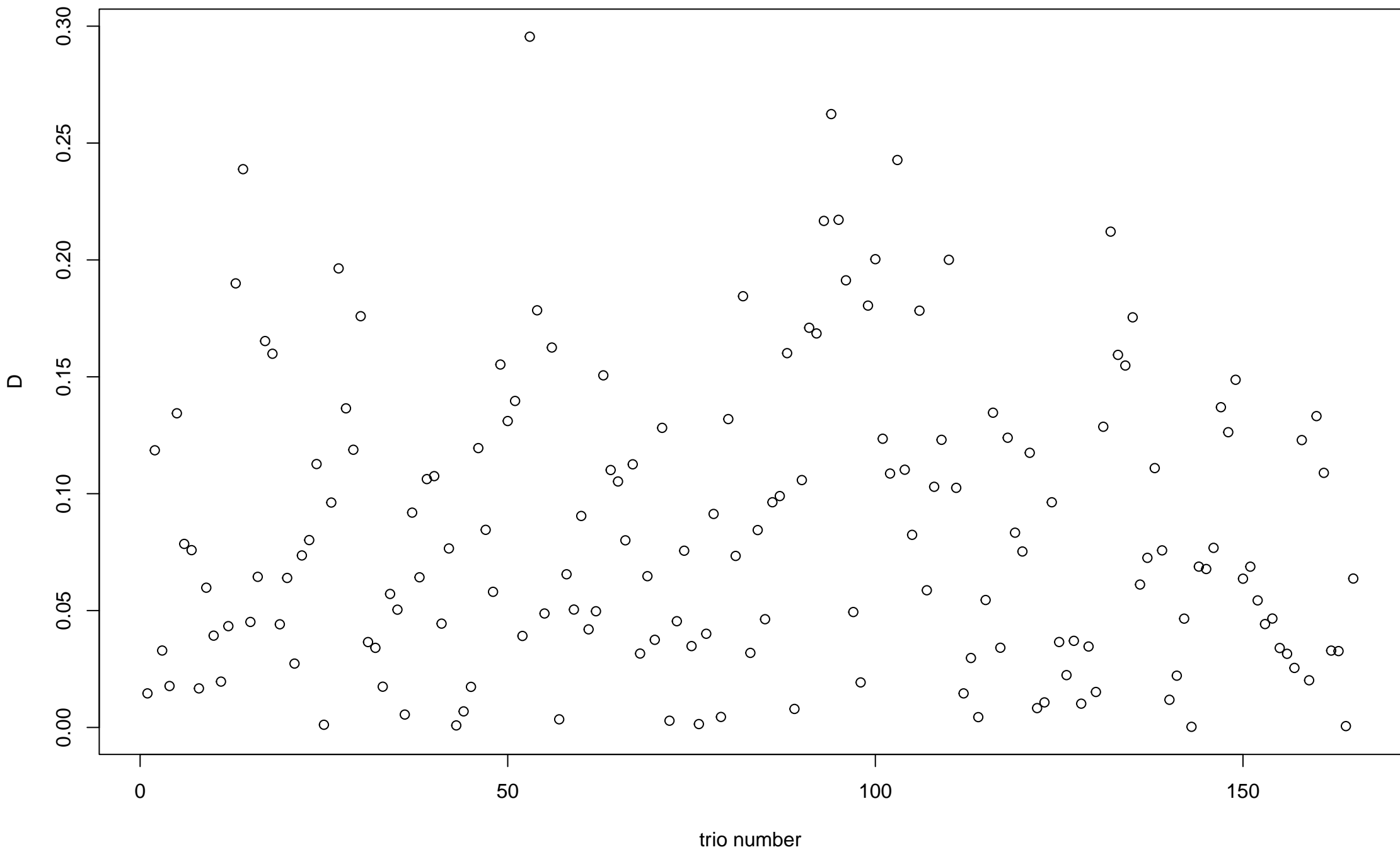

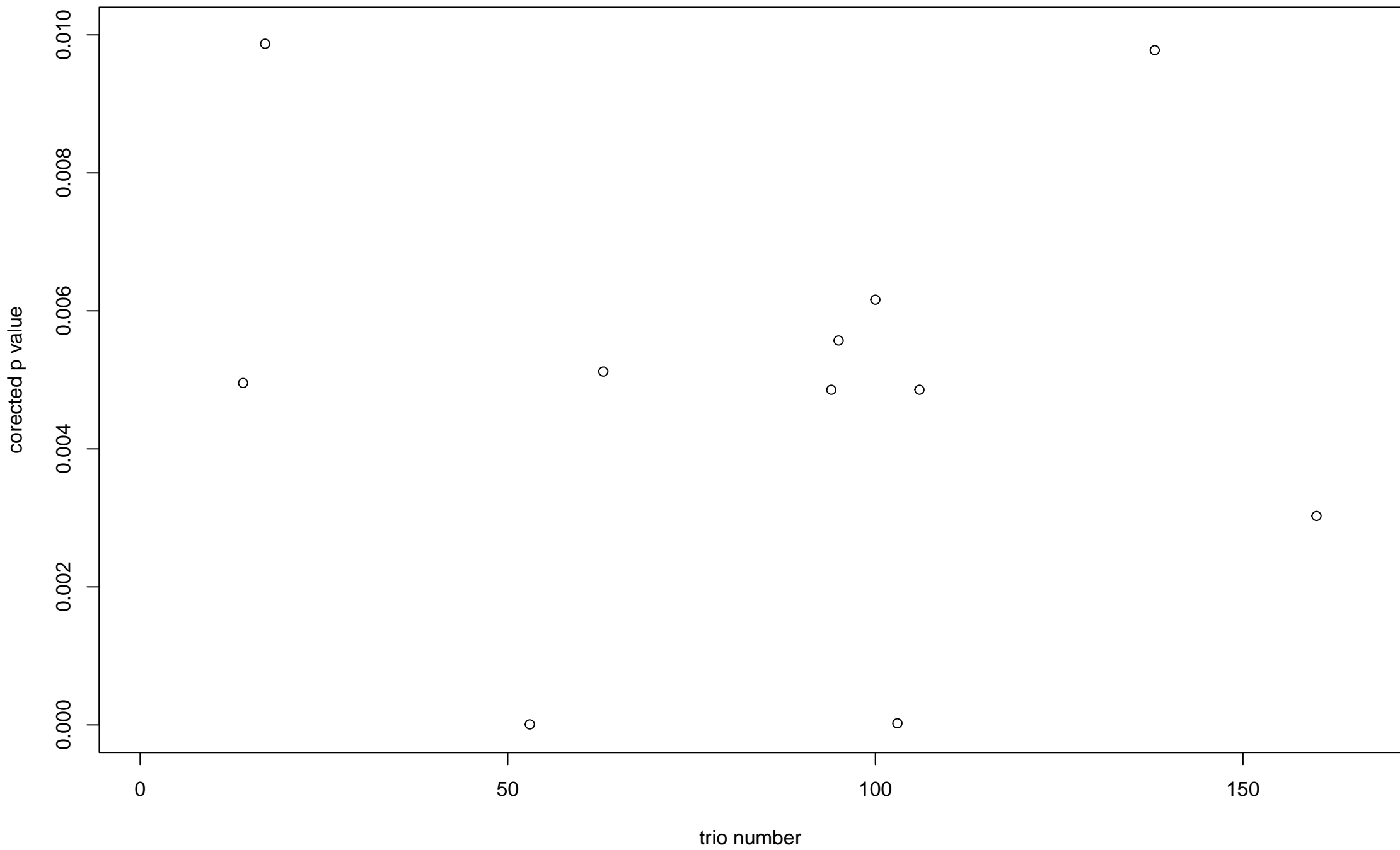

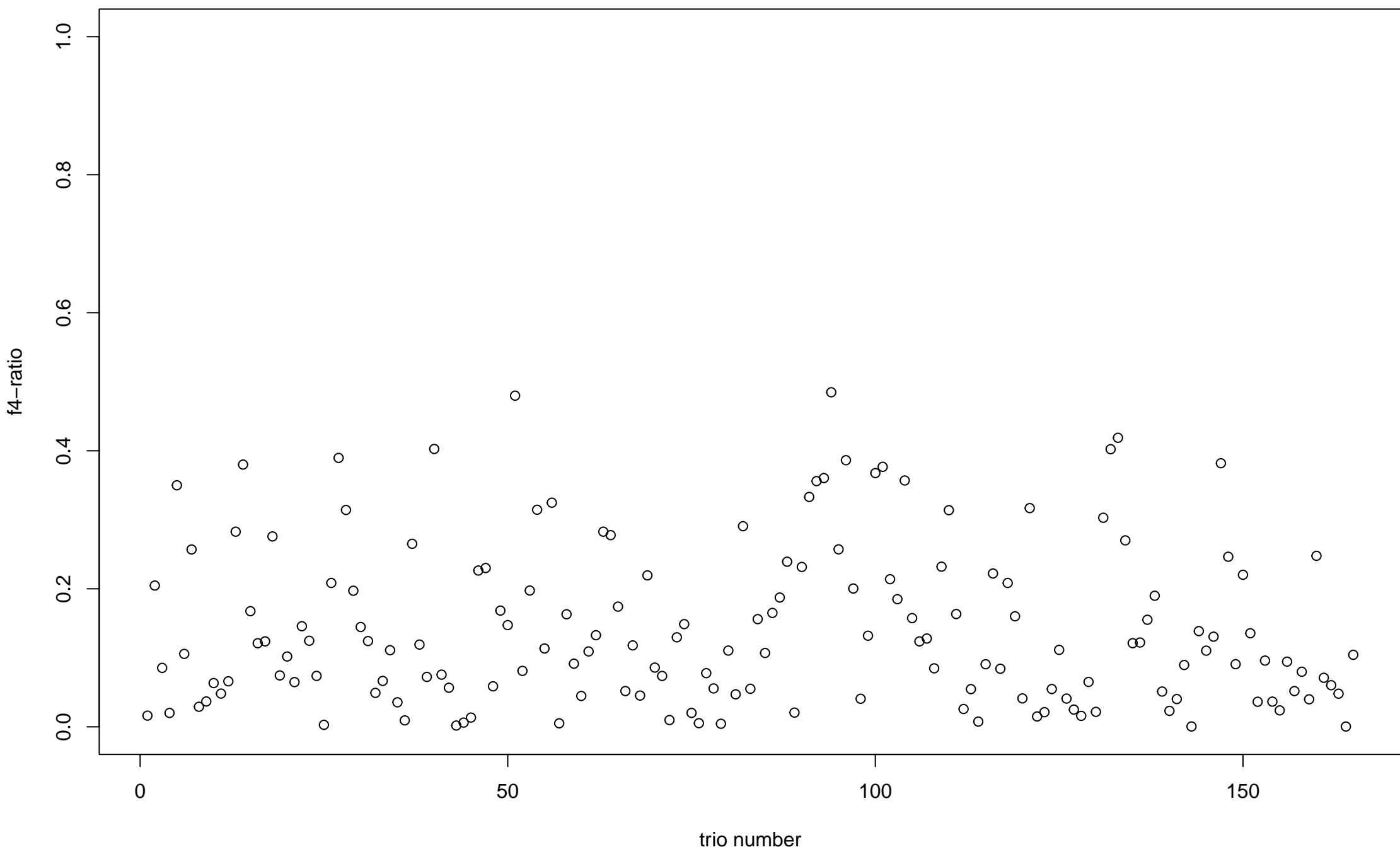

Supplement: Supplementary file 1 [file plants-13-01987-s001.zip › Figure S3_D_BBAA_GF_adjust.p.value.pdf]
